# Plant-Symbiotic Fungi as Chemical Engineers: Multi-Genome Analysis of the Clavicipitaceae Reveals Dynamics of Alkaloid Loci

**DOI:** 10.1371/journal.pgen.1003323

**Published:** 2013-02-28

**Authors:** Christopher L. Schardl, Carolyn A. Young, Uljana Hesse, Stefan G. Amyotte, Kalina Andreeva, Patrick J. Calie, Damien J. Fleetwood, David C. Haws, Neil Moore, Birgitt Oeser, Daniel G. Panaccione, Kathryn K. Schweri, Christine R. Voisey, Mark L. Farman, Jerzy W. Jaromczyk, Bruce A. Roe, Donal M. O'Sullivan, Barry Scott, Paul Tudzynski, Zhiqiang An, Elissaveta G. Arnaoudova, Charles T. Bullock, Nikki D. Charlton, Li Chen, Murray Cox, Randy D. Dinkins, Simona Florea, Anthony E. Glenn, Anna Gordon, Ulrich Güldener, Daniel R. Harris, Walter Hollin, Jolanta Jaromczyk, Richard D. Johnson, Anar K. Khan, Eckhard Leistner, Adrian Leuchtmann, Chunjie Li, JinGe Liu, Jinze Liu, Miao Liu, Wade Mace, Caroline Machado, Padmaja Nagabhyru, Juan Pan, Jan Schmid, Koya Sugawara, Ulrike Steiner, Johanna E. Takach, Eiji Tanaka, Jennifer S. Webb, Ella V. Wilson, Jennifer L. Wiseman, Ruriko Yoshida, Zheng Zeng

**Affiliations:** 1 Department of Plant Pathology, University of Kentucky, Lexington, Kentucky, United States of America; 2 Forage Improvement Division, The Samuel Roberts Noble Foundation, Ardmore, Oklahoma, United States of America; 3 Eastern Kentucky University, Richmond, Kentucky, United States of America; 4 AgResearch Laboratory, School of Biological Sciences, University of Auckland, Auckland, New Zealand; 5 Statistics Department, University of Kentucky, Lexington, Kentucky, United States of America; 6 Computer Science Department, University of Kentucky, Lexington, Kentucky, United States of America; 7 Institute of Plant Biology and Biotechnology, University of Muenster, Muenster, Germany; 8 Division of Plant and Soil Sciences, West Virginia University, Morgantown, West Virginia, United States of America; 9 Grasslands Research Centre, AgResearch, Palmerston North, New Zealand; 10 Department of Chemistry and Biochemistry, Stephenson Research and Technology Center, University of Oklahoma, Norman, Oklahoma, United States of America; 11 The John Bingham Laboratory, National Institute of Agricultural Botany, Cambridge, United Kingdom; 12 Institute of Molecular BioSciences, Massey University, Palmerston North, New Zealand; 13 Texas Therapeutics Institute, University of Texas Health Science Center, Houston, Texas, United States of America; 14 Forage-Animal Production Research Unit, United States Department of Agriculture Agricultural Research Service, Lexington, Kentucky, United States of America; 15 Toxicology and Mycotoxin Research Unit, Richard B. Russell Research Center, United States Department of Agriculture Agricultural Research Service, Athens, Georgia, United States of America; 16 Institute of Bioinformatics and Systems Biology, Helmholtz Zentrum München (GmbH), Neuherberg, Germany; 17 Invermay Agricultural Centre, Mosgiel, New Zealand; 18 Institut fuer Pharmazeutische Biologie, Universitaet Bonn, Bonn, Germany; 19 Institute of Integrative Biology, ETH Zürich, Zürich, Switzerland; 20 College of Pastoral Agriculture Science and Technology, Lanzhou University, Lanzhou, China; 21 Institute of Livestock and Grassland Science, National Agriculture and Food Research Organization (NARO), Nasushiobara, Tochigi, Japan; 22 Ishikawa Prefectural University, Suematsu, Nonoichi, Ishikawa, Japan; Duke University Medical Center, United States of America

## Abstract

The fungal family Clavicipitaceae includes plant symbionts and parasites that produce several psychoactive and bioprotective alkaloids. The family includes grass symbionts in the epichloae clade (*Epichloë* and *Neotyphodium* species), which are extraordinarily diverse both in their host interactions and in their alkaloid profiles. Epichloae produce alkaloids of four distinct classes, all of which deter insects, and some—including the infamous ergot alkaloids—have potent effects on mammals. The exceptional chemotypic diversity of the epichloae may relate to their broad range of host interactions, whereby some are pathogenic and contagious, others are mutualistic and vertically transmitted (seed-borne), and still others vary in pathogenic or mutualistic behavior. We profiled the alkaloids and sequenced the genomes of 10 epichloae, three ergot fungi (*Claviceps* species), a morning-glory symbiont (*Periglandula ipomoeae*), and a bamboo pathogen (*Aciculosporium take*), and compared the gene clusters for four classes of alkaloids. Results indicated a strong tendency for alkaloid loci to have conserved cores that specify the skeleton structures and peripheral genes that determine chemical variations that are known to affect their pharmacological specificities. Generally, gene locations in cluster peripheries positioned them near to transposon-derived, AT-rich repeat blocks, which were probably involved in gene losses, duplications, and neofunctionalizations. The alkaloid loci in the epichloae had unusual structures riddled with large, complex, and dynamic repeat blocks. This feature was not reflective of overall differences in repeat contents in the genomes, nor was it characteristic of most other specialized metabolism loci. The organization and dynamics of alkaloid loci and abundant repeat blocks in the epichloae suggested that these fungi are under selection for alkaloid diversification. We suggest that such selection is related to the variable life histories of the epichloae, their protective roles as symbionts, and their associations with the highly speciose and ecologically diverse cool-season grasses.

## Author Summary

The fungal family, Clavicipitaceae, includes “ergot” fungi that parasitize ears of cereals and have historically caused mass poisonings, as well as “epichloae,” which are symbionts of grasses. Many epichloae are mutualistic symbionts, but some are pathogenic, and others have both mutualistic and pathogenic characteristics. Most Clavicipitaceae produce “alkaloids,” small molecules that deter insects, livestock, and wildlife from feeding on the fungus or plant. Epichloae protect their hosts with diverse alkaloids belonging to four chemical classes. After sequencing the entire DNA contents (“genomes”) of ten epichloae, three ergot fungi, and two relatives, we compared their “clusters” of genes for alkaloid biosynthesis. In the epichloae, these clusters contained extraordinarily large blocks of highly repetitive DNA, which promote gene losses, mutations, and even the evolution of new genes. These repeat blocks account for the exceptionally high alkaloid diversity in the epichloae and may relate to the ecological diversity of these symbiotic fungi.

## Introduction

Alkaloids play key roles in plant ecology by targeting the central and peripheral nervous systems of invertebrate and vertebrate animals, affecting their behavior, eliciting toxicoses, and reducing herbivory [1]. Alkaloids are very common in plants as well as certain plant-associated fungi, particularly those in the family Clavicipitaceae. Plant parasites such as *Claviceps* species often produce high levels of ergot alkaloids or indole-diterpenes, probably to defend their resting and overwintering structures (commonly called ergots) [2], [3]. A closely related group of fungi, the epichloae (*Epichloë* and *Neotyphodium* species) live as systemic symbionts of grasses, and produce a wide array of alkaloids that combat various herbivorous animals, a key determinant of mutualism in many grass-endophyte symbioses [4], [5].

**Figure 1:**
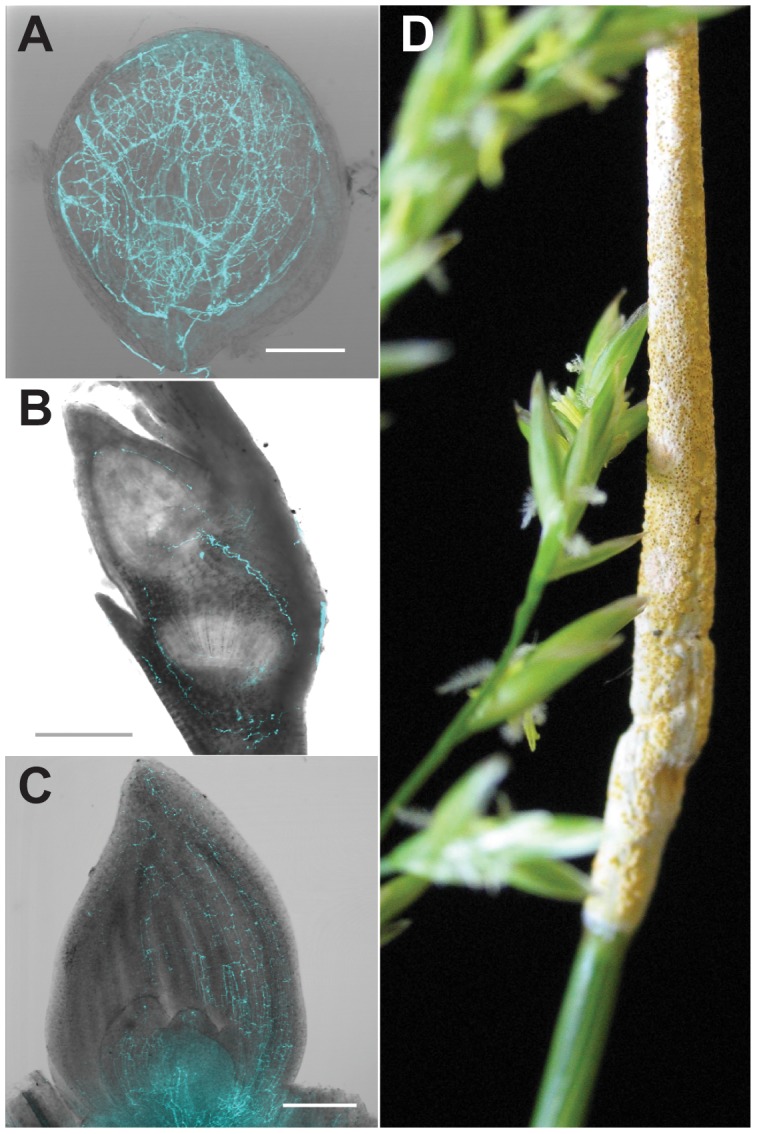
Symbiosis of meadow fescue with *Epichloë festucae*, a heritable symbiont. Single optical slice confocal micrographs of *E. festucae* expressing enhanced cyan-fluorescent protein were overlain with DIC bright field images of (A) ovules (bar = 100 µm), (B) embryos (bar = 200 µm), and (C) shoot apical meristem and surrounding new leaves (bar = 200 µm). (D) Asymptomatic (left) and “choked” (right) inflorescences simultaneously produced on a single grass plant infected with a single *E. festucae* genotype. Vertical (seed) transmission of the symbiont occurs via the asymptomatic inflorescence, whereas the choked inflorescence bears the *E. festucae* fruiting structure (stroma), which produces sexually derived spores (ascospores) that mediate horizontal transmission.

Fungi of family Clavicipitaceae are generally biotrophs that grow in invertebrates, fungi, or plants. The major clade of plant-associated Clavicipitaceae [6] includes mutualistic symbionts as well as plant pathogens, many of which produce alkaloids with diverse neurotropic effects on vertebrate and invertebrate animals with important implications for human health, agriculture and food security [7], [8]. Most species of plant-associated Clavicipitaceae grow in or on grasses, but the group also includes systemic parasites of sedges or other plants, and heritable symbionts of morning glories [9]. The plant-associated Clavicipitaceae have very high chemotypic diversity, ecological significance [10], and agricultural impact [11]. Many produce abundant alkaloids such as ergot alkaloids and indole-diterpenes, which have potent neurotropic activities in mammals. The ergot alkaloids are named for the ergot fungi (*Claviceps* species), which are infamous for causing mass poisonings throughout much of human history, although ergot alkaloids also have numerous pharmaceutical uses [7], [12]–[14]. In contrast to the *Claviceps* species, the epichloae (*Epichloë* or *Neotyphodium* species) are systemic and often heritable, mutualistic symbionts of cool-season grasses (Poaceae, subfamily Poöideae)(Figure 1) [4]. Epichloae have diverse alkaloid profiles, and in addition to ergot alkaloids or indole-diterpenes, many produce lolines or peramine, which help to protect their grass hosts from insects [15], [16] and possibly other invertebrates [17].

The activities of alkaloids in animal nervous systems relates to their chemical similarities to biogenic amines [1]. Although poisoning of humans by alkaloids of clavicipitaceous fungi is now rare, toxicity to livestock is frequently observed [18]–[20]. Morning glories such as *Ipomoeae asarifolia* cause toxicity to livestock on ranges in Brazil, probably due to alkaloids produced by symbiotic *Periglandula ipomoeae* [9], [21]. Indole-diterpene or ergot alkaloids produced by epichloae in wild and cultivated grasses also can cause livestock toxicosis [7], [22]. For example, in 1993, losses to pastured U.S. beef production were estimated at $600 million due to widespread plantings of tall fescue symbiotic with ergot-alkaloid-producing strains of *Neotyphodium coenophialum* [23]. In addition to chemotypic variation [24], the epichloae also exhibit an extraordinary variety of host-interactions, whereby some are pathogenic and contagious, others are mutualistic and vertically transmitted (heritable), and others have both mutualistic and pathogenic characteristics [4], [25]. Relative benefits of epichloae and their alkaloids to host grasses are related to variations in life history [26], [27] and ecological contexts [28], [29], which may well explain why they have evolved such chemotypic diversity.

**Figure 2:**
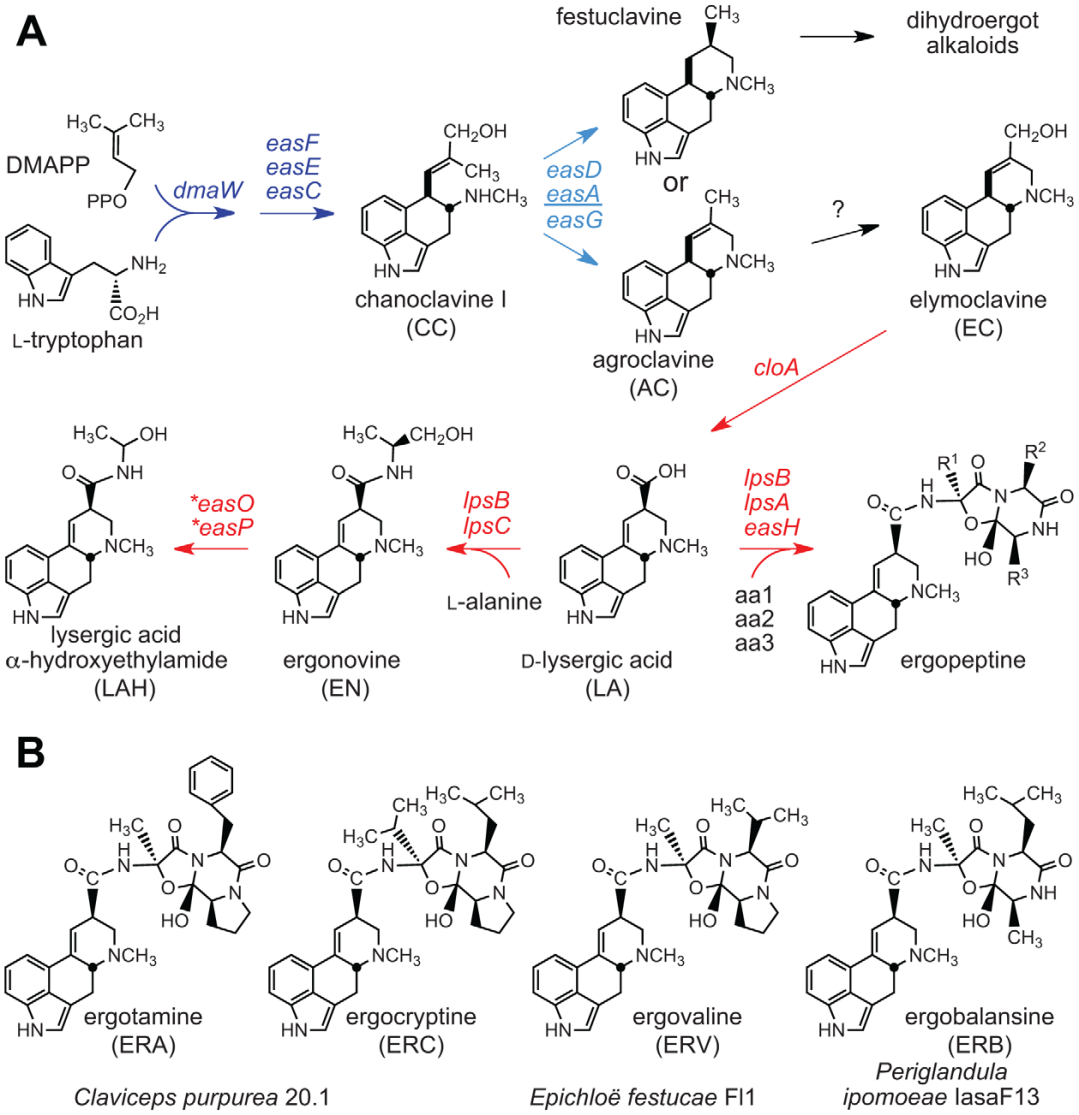
Ergot alkaloids and summary of biosynthesis pathway. (A) Ergoline alkaloid biosynthesis pathways in the Clavicipitaceae. Arrows indicate one or more steps catalyzed by products of genes indicated. Arrows and genes in blue indicate steps in synthesis of the first fully cyclized intermediate (skeleton). Variation in the *easA* gene (underlined) determines whether the ergoline skeleton is festuclavine or agroclavine. Arrows and genes in red indicate steps in decoration of the skeleton to give the variety of ergolines in the Clavicipitaceae. Asterisks indicate genes newly discovered in the genome sequences of *C. paspali*, *N. gansuense* var. *inebrians* and *P. ipomoeae*. (B) Ergopeptines produced by strains in this study.

Even within an alkaloid class, structural variations can profoundly affect pharmacological spectra, as reflected for example in the diverse uses of ergot alkaloids in medicine [14], [30](Figure 2). Ergonovine ( = ergometrine) was long used to aid in childbirth, ergotamine is used for migraines, and, in recent years, 2-bromonated ergocryptine (bromocriptine) has been adopted for treatment of numerous disorders of the central nervous system, such as Parkinsonism and pituitary gland adenomas. In contrast, lysergic acid diethylamide (LSD), a semisynthetic ergot alkaloid originally developed as an antidepressant, is the most potent hallucinogen known [31], and was a major factor in the drug culture of the 1960's and 1970's. Historic episodes of mass poisoning in humans have resulted from contamination of grains with ergots (the resting structures of *Claviceps* species) [32], and the effects vary depending on which alkaloids are present. Symptoms range from the disfiguring dry gangrene of St. Anthony's fire to convulsions and hallucinations such as those associated with the Salem witch trials [33]. For example, outbreaks of convulsive ergotism in India in the late 1970's were due to *Claviceps fusiformis* producing mainly elymoclavine [34], while Ethiopia experienced a gangrenous ergotism outbreak in 1978 caused by *C. purpurea* producing ergopeptines [35].

**Figure 3:**
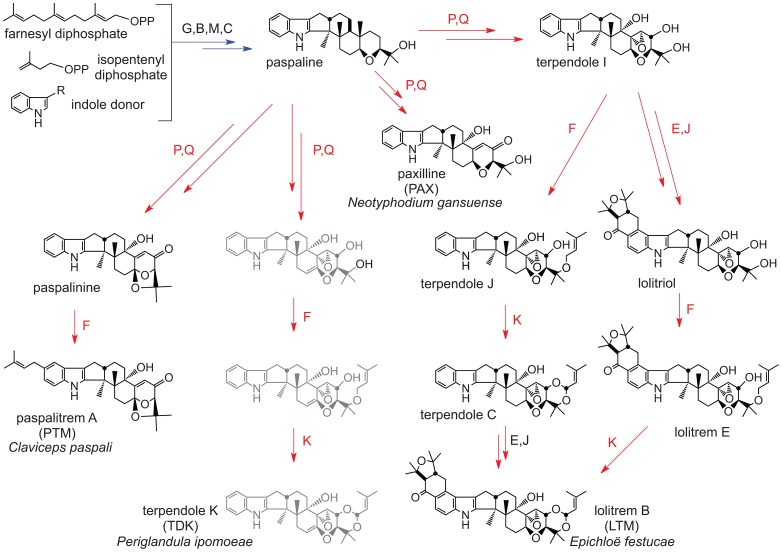
Summary of indole-diterpene biosynthesis pathway. Arrows indicate one or more steps catalyzed by products of the genes indicated, where each *idt/ltm* gene is designated by its final letter (*G = idtG/ltmG*, etc.). Arrows and genes in blue indicate steps in synthesis of the first fully cyclized intermediate (paspaline). Arrows and genes in red indicate steps in decoration of paspaline to give the variety of indole-diterpenes in the Clavicipitaceae. Structures shown in gray are not yet verified.

**Figure 4:**
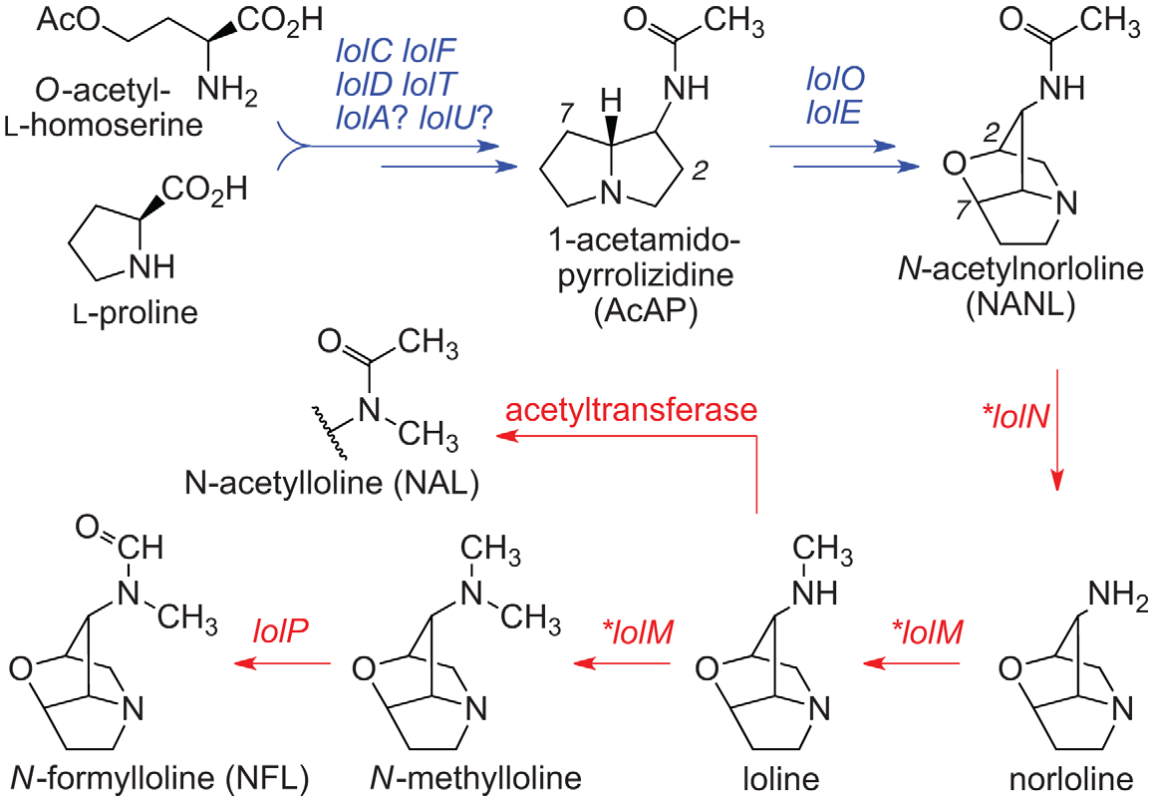
Summary of loline alkaloid-biosynthesis pathway. Arrows indicate one or more steps catalyzed by products of the genes indicated. Arrows and genes in blue indicate steps in synthesis of the first fully cyclized intermediate (NANL). Arrows and genes in red indicate steps in modification of NANL to give the variety of lolines found in the epichloae. Asterisks indicate *LOL* genes that were newly discovered in the genome sequence of *E. festucae* E2368.

Other alkaloids produced by Clavicipitaceae variously present hazards or benefits to agriculture. The indole-diterpenes (Figure 3) represent a broad diversity of bioactive compounds that exhibit mammalian and insect toxicity through activation of various ion channels [36], [37]. Livestock afflicted with indole-diterpene toxicity display symptoms of ataxia and sustained tremors [20]. For example, Paspalum staggers is caused by paspalitrems produced by *Claviceps paspali* and *Claviceps cynodontis* on seed-heads of dallisgrass (*Paspalum dilatatum*) and Bermuda grass (*Cynodon dactylon*), respectively [3], and common strains of *Neotyphodium lolii* symbiotic with perennial ryegrass (*Lolium perenne*) produce lolitrems, which cause ryegrass staggers [20]. In contrast, lolines (Figure 4) and peramine produced by many endophytic epichloae in forage grasses have not been linked to any toxic symptoms in grazing mammals, but instead provide potent protection from herbivorous insects [15], [16].

The discoveries of individual genes involved in biosynthetic pathways for each of the four alkaloid classes [16], [38]–[40] has led to elucidation of clusters of biosynthesis genes for ergot alkaloids (*EAS*) in *C. purpurea* [41], lolines (*LOL*) in *Neotyphodium uncinatum* [42], and lolitrems (a group of indole-diterpenes, *IDT/LTM*) in *Neotyphodium lolii* [43], as well as characterization of the *perA* gene of *Epichloë festucae* [16]. The identification of these genetic loci, elucidation of structural diversity within each alkaloid class, and new technologies for high-throughput DNA sequencing together provide an outstanding opportunity to investigate the genome dynamics governing chemotypic variation in fungi with diverse life histories and ecological functions. To that end, we sequenced genomes and compared alkaloid locus structures of 15 plant-associated Clavicipitaceae, including 10 epichloae, three *Claviceps* species, the nonculturable morning glory symbiont *Periglandula ipomoeae*, and the bamboo witch's broom pathogen *Aciculosporium take* (Table S1). We report that the alkaloid loci tend to be arranged with genes for conserved early pathway steps in their cores, and peripheral genes that vary in presence or absence, or in sequence, to diversify structures within each alkaloid class. Transposon-derived repeats, miniature inverted repeat transposable elements (MITEs), and telomeres were often associated with unstable loci or the variable peripheral genes, and were especially common in alkaloid clusters of the epichloae. We suggest that structures of the alkaloid loci, including distributions of repeat blocks, reflect selection on these fungi for niche adaptation.

## Results

### Genome sequences

**Figure 5:**
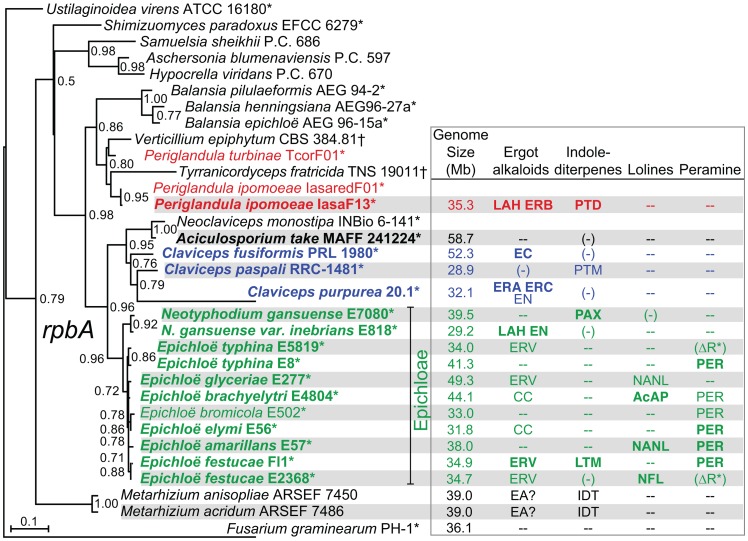
Phylogenies of *rpbA* from sequenced isolates and other Clavicipitaceae. The phylogenetic tree is based on nucleotide alignment for a portion of the RNA polymerase II largest subunit gene, *rpbA*. This tree is rooted with *Fusarium graminearum* as the outgroup. Epichloae are indicated in green, *Claviceps* species are indicated in blue, *Periglandula* species are indicated in red, and *Aciculosporium take* is in black. Species for which genomes were sequenced in this study are shown in bold type, and asterisks indicate plant-associated fungi. Alkaloids listed are the major pathway end-products predicted from the genome sequences, abbreviated as shown in Figure 2, Figure 3, Figure 4. Other abbreviations: (−) = some genes or remnants present, but not predicted to make alkaloids of this class, – = no genes present for this alkaloid class, EA = ergot alkaloids may be produced; IDT = indole-diterpenes may be produced, (ΔR*) = deletion of terminal reductase domain of *perA*.

Clusters of genes have been identified for the four alkaloid biosynthesis classes [16], [41]–[43], but in the absence of complete genome sequences it was unknown if the clusters had been fully characterized for any known producers in the Clavicipitaceae. Therefore, we sequenced 15 genomes of diverse species in the family with various alkaloid profiles (Figure 5, Table 1). The genomes were primarily sequenced by shotgun pyrosequencing, but paired-end and mate-pair reads were used to scaffold several assemblies. Notably, adding mate-pair pyrosequencing of *C. purpurea* DNA resulted in a 186-supercontig (scaffold) assembly of 32.1 Mb, and adding end-sequencing of fosmid clones *E. festucae* Fl1 DNA resulted in a 170-supercontig assembly of 34.9 Mb. Annotated genome sequences have been posted at www.endophyte.uky.edu, and (for *C. purpurea* 20.1) at http://www.ebi.ac.uk/ena/data/view/Project:76493, and GenBank and EMBL project numbers are listed in Table S2. Assembled genome sizes among the sequenced strains varied 2-fold from 29.2 to 58.7 Mb, with wide ranges even within the genera *Claviceps* (31–52.3 Mb) and *Epichloë* (29.2–49.3 Mb) (Table 1). The majority of genome size variation was due to repeat sequences, which ranged from 4.7–56.9% overall (excluding *P. ipomoeae* from consideration because contigs that lacked coding sequences may have been filtered from that assembly), and from 13.7–44.9% repeat DNA among the epichloae (Table 2). Also, the average GC contents of repeat sequences varied widely, from 22% in *C. fusiformis* PRL 1980 to 50% in *C. purpurea* 20.1 (Table 3). The sums of coding sequence lengths were estimated from *ab initio* gene predictions with FGENESH, and ranged from 9.4 Mb in *A. take* MAFF-241224 to 15.9 Mb in *P. ipomoeae* IasaF13 (Table 2). Most of the epichloae had approximately 11 Mb of coding sequence, with the exception of *E. glyceriae* E277, which had 14.9 Mb of coding sequence. Gene contents were not correlated with genome size, and although *A. take* had the largest genome at 58.7 Mb, it had the least coding sequence at 9.4 Mb.

**Table 1:** Genome sequencing statistics for plant-associated Clavicipitaceae.^a^

Species	Strain	Genome assembly size (Mb)^b^	x-cov	Ctg no.	Ctg N50	PE & MP frag. (kb)	Sc no.	Sc N50	N50 Sc no.
*Aciculosporium take*	MAFF-241224	58.7	32.4	2890	40 562	none	n/a	n/a	n/a
*Claviceps fusiformis*	SD58	52.3	34.9	6178	20 136	none	n/a	n/a	n/a
*Claviceps paspali*	RRC–1481	28.9	54.4	2152	26 979	none	n/a	n/a	n/a
*Claviceps purpurea*	20.1	32.1	39.0	1878	44 165	3.2	186	433 700	26
*Epichloë amarillans*	E57	38.0	27.7	1871	49 298	none	n/a	n/a	n/a
*Epichloë brachyelytri*	E4804	44.1	24.6	4542	20 998	none	n/a	n/a	n/a
*Epichloë elymi*	E56	31.8	25.6	5206	32 484	0.6	2117	39 522	202
*Epichloë festucae*	E2368	34.7	27.8	1316	87 544	3.8	813	163 634	58
*Epichloë festucae*	Fl1	34.9	52.3	1277	84 986	2.0, 36.1	170	436 939	24
*Epichloë glyceriae*	E277	49.3	27.7	2658	42 649	none	n/a	n/a	n/a
*Epichloë typhina*	E8	41.3	21.2	2253	42 567	none	n/a	n/a	n/a
*Epichloë typhina*	E5819	34.0	28.6	2072	36 475	none	n/a	n/a	n/a
*Neotyphodium gansuense*	E7080	39.5	41.5	1307	56 237	none	n/a	n/a	n/a
*N. gansuense* var. *inebrians*	E818	29.2	53.0	3302	22 140	none	n/a	n/a	n/a
*Periglandula ipomoeae*	IasaF13	35.3	19.8	869	66 619	none	n/a	n/a	n/a

a Abbreviations: Ctg = contig; PE = paired end; MP = mate pair; Sc = scaffold (i.e., supercontig); x-cov. = fold coverage of sequence. N50 is defined as the minimum length of the largest contigs or scaffolds (as specified) that together contain 50% of the genome assembly.

b Size based on total scaffold length for *C. purpurea* 20.1, *E. festucae* E2368, and *E. festucae* Fl1, and total length of large (≥500 bp) contigs for the others.

**Table 2:** Genic and repeat DNA contents of sequenced genomes.^a^

Organism	Strain	MT	Genome Assembly Length (Mb)^b^	Total Genic (Mb)	Total CDS (Mb)	% CDS	% non-Rpt-IG	% Rpt
*Aciculosporium take*	MAFF-241224	B	58.7	14.0	9.4	16.0	20.1	56.9
*Claviceps fusiformis*	PRL 1980	B^c^	52.3	19.2	11.9	22.7	22.1	45.7
*C. paspali*	RRC-1481	B	28.9	14.4	10.0	34.7	33.3	17.5
*C. purpurea*	20.1	A	31.0	17.5	12.2	39.4	40.2	4.7
*Epichloë amarillans*	E57	B	38.0	15.9	10.9	28.7	21.7	38.8
*E. brachyelytri*	E4804	B	44.1	16.5	10.9	24.7	21.3	44.9
*E. elymi*	E56	A	31.8	15.4	10.8	33.9	27.5	25.7
*E. festucae*	E2368	A	34.4	15.9	11.1	32.5	25.9	29.2
*E. festucae*	Fl1	B	34.2	15.6	10.9	31.9	28.7	26.9
*E. glyceriae*	E277	A	49.3	20.4	14.9	30.3	22.0	37.6
*E. typhina*	E8	A	41.2	15.5	10.3	30.9	27.5	29.9
*E. typhina*	E5819	A	34.0	15.2	10.5	25.1	23.8	41.6
*Neotyphodium gansuense*	E7080	B	39.5	16.6	11.7	29.7	22.7	36.1
*N. gansuense* var. *inebrians*	E818	A	29.2	16.1	11.2	38.5	32.5	13.7
*P. ipomoeae* d	IasaF13	A^c^	35.3	22.5	15.9	45.0	36.3	0.1

a Abbreviations: CDS = coding sequence, MT = mating type, non-Rpt-IG = nonrepetitive intergenic DNA, Rpt = repetitive DNA.

b Based on total of contigs ≥500 bp. These sizes differ slightly from total scaffold lengths given in Table 1 for *C. purpurea* 20.1, *E. festucae* E2368, and *E. festucae* Fl1.

c *C. fusiformis* PRL 1980 mating type genes include *mtBA* and *mtAC*. *P. ipomoeae* IasaF13 mating type genes *mtAA* and *mtAC* appear to have premature stop codons.

d Statistics for *P. ipomoeae* are tentative because the assembly was filtered by selecting only contigs containing tBLASTx matches to genome sequences from the other Clavicipitaceae.

**Table 3:** GC proportions in genic and repeat DNA of sequenced genomes.^a^

Organism	Strain	Genome GC	CDS GC	non-Rpt-IG GC	Rpt GC
*Aciculosporium take*	MAFF-241224	0.40	0.59	0.49	0.31
*Claviceps fusiformis*	PRL 1980	0.37	0.55	0.45	0.22
*C. paspali*	RRC-1481	0.48	0.58	0.49	0.23
*C. purpurea*	20.1	0.52	0.55	0.50	0.50
*Epichloë amarillans*	E57	0.44	0.55	0.49	0.32
*E. brachyelytri*	E4804	0.40	0.54	0.47	0.28
*E. elymi*	E56	0.47	0.55	0.49	0.33
*E. festucae*	E2368	0.44	0.55	0.49	0.28
*E. festucae*	Fl1	0.44	0.55	0.47	0.27
*E. glyceriae*	E277	0.45	0.54	0.49	0.36
*E. typhina*	E8	0.42	0.55	0.48	0.28
*E. typhina*	E5819	0.43	0.55	0.48	0.23
*Neotyphodium gansuense*	E7080	0.44	0.54	0.48	0.32
*N. gansuense* var. *inebrians*	E818	0.47	0.54	0.47	0.27
*P. ipomoeae* b	IasaF13	0.51	0.53	0.50	0.42

a Abbreviations: CDS = coding sequence, GC = proportion of sequence that is G or C, non-Rpt-IG = nonrepetitive intergenic DNA, Rpt = repetitive DNA.

b Statistics for *P. ipomoeae* are tentative because the assembly was filtered by selecting only contigs containing tBLASTx matches to genome sequences from the other Clavicipitaceae.

### Phylogenetic relationships

Phylogenetic analysis of aligned partial coding sequences for the RNA polymerase II largest subunit (*rpbA*) for all of the sequenced isolates, together with related fungi for which the sequence data are available (Figure 5), supported the relationships previously indicated for subsets of these fungi [44], [45]. The sequenced strains were contained in a clade that mainly included Clavicipitaceae associated with the plant families, Poaceae (grasses), and Convolvulaceae (morning glories). These had more distant relationships to the Clavicipitaceae associated with insects. The *Epichloë* and *Neotyphodium* species grouped in a single clade (epichloae), and until recently the sexual species were classified in genus *Epichloë*, and those with no known sexual state were classified in form genus *Neotyphodium* [25]. (This was in accord with the dual naming system for fungi, formerly specified in the botanical code of nomenclature.) The sister clade to the epichloae included the *Claviceps*, *Aciculosporium* and *Neoclaviceps* species. Outside of this clade grouped other plant associates and insect associates, including two *Metarhizium* species for which there are recently published genome sequences [46]. *Metarhizium* species are well-known insect pathogens, although some strains of *Metarhizium anisopliae* have recently been shown to be associated with plant rhizospheres [47].

Phylogenies of partial coding sequences for *rpbA* and two other housekeeping genes, *rpbB* (encoding RNA polymerase II second-largest subunit) and *tefA* (encoding translation elongation factor 1-α) (Figure S1) were compared by the Shimodaira-Hasegawa test (Table S3). The *rpbA* phylogeny was congruent with the *rpbB* phylogeny, but the *tefA* phylogeny was significantly incongruent with those of *rpbA* and *rpbB*. The *tefA* tree had a very different placement of *M. anisopliae* than did the other two phylogenies. Nevertheless, all three gene trees were in agreement with respect to the grouping of the epichloae in a single clade, with a sister clade that included *Claviceps* species and *A. take*. All trees also supported a relationship of *P. ipomoeae* (and, for *rbpA*, *P. turbinae*) with the fungal parasite, *Verticillium epiphytum*. Inclusion of another fungal parasite, *Tyranicordyceps fratricida*m, with *Periglandula* spp. and *V. epiphytum* was supported by the *rpbA* and *rpbB* trees, and not significantly contradicted by the *tefA* tree.

### Alkaloid profiles

Plant-associated Clavicipitaceae generally produce alkaloids most consistently in association with host plants [16], [42], [43], [48], [49], so samples of plant material symbiotic with several epichloae were profiled for combinations of ergot alkaloids, indole-diterpenes, lolines and peramine, depending on which gene clusters were identified in the sequenced genomes. Symbiotic material was available for *E. amarillans* E57, *E. elymi* E56, *E. festucae* E2368 and Fl1, *E. glyceriae* E2772, *E. typhina* E8, *N. gansuense* E7080, *N. gansuense* var. *inebrians* E818, and *N. uncinatum* E167, and in limited amounts (sufficient for a loline alkaloid analysis) from *E. brachyelytri* E4804. Leaves and seeds of morning glory symbiotic with *P. ipomoeae* IasaF13 were assayed for ergot alkaloids and indole-diterpenes. Ergot alkaloids also were analyzed from ergots of *C. purpurea* 20.1, and the ergot alkaloid profile of *C. fusiformis* PRL 1980 is well established [50]. No infected plant material was available to assay the alkaloid profile of *E. typhina* E5819, and no ergots of *C. paspali* RRC-1481 were available. Alkaloid profiles listed in Table 4 indicated both interspecific and intraspecific variation.

**Table 4:** Alkaloid profiles of sequenced isolates.^a^

	Strain													
Alkaloid	*C* *f* *u*	*C* *p* *a*	*C* *p* *u*	*E* *a* *m*	*E* *b* *e*	*E* *e* *l*	*E* *f*1	*E* *f*2	*E* *g* *l*	*E* *t*8	*N* *g* *a*	*N* *g* *i*	*N* *u* *n*	*P* *i* *p*
**Ergot alkaloids:**														
Chanoclavine (CC)	+		(+)		nt	+	(+)	(−)	(−)			+		+
Agroclavine	+		(+)			−	(+)	(−)	(−)			(+)		+
Elymoclavine (EC)	+		(+)			−	(+)	(−)	(−)			(+)		(+)
Lysergic acid	−		(+)			−	(+)	(−)	(−)			(+)		(+)
Lysergic acid amide	−		nt			−	−	−	−			+		+
Ergonovine (EN)	−		nt			−	−	−	−			+		+
Lysergic acid α-hydroxyethylamide (LAH)	−					−	−	−	−			+		+
Ergopeptine	−		ERA ERC			−	ERV	(−)	(−)			−		ERB
**Indole-diterpenes:** b														
Paspaline		nt					+				+			+
Paspaline B		nt					+				+			+
13-Desoxypaxilline							+				+			+
Paxilline (PAX)							−				+			+
IDT-436							−				−			+
Terpendole E							(+)				−			+
Terpendole I							(+)				−			+
Terpendole J							(+)				−			+
Terpendole C							+				−			+
Terpendole K (TDK)							−				−			+
Terpendole M							−				−			+
Terpendole N							−				−			+
Terpendole A							−				−			+
Lolicine A							+				−			−
Loliline							+				−			−
Lolitrem E							+				−			−
Lolitrem B (LTM)							+				−			−
**Lolines:**														
Acetamidopyrrolizidine (AcAP)				(+)	+			(+)	(+)				+	
*N*-Acetylnorloline (NANL)				+	−			+	+				+	
Loline				−	−			+	−				+	
*N*-Acetylloline				−	−			+	−				+	
*N*-Methylloline				−	−			+	−				+	
*N*-Formylloline (NFL)				−	−			+	−				+	
**Peramine (PER)**				+	nt	+	+	−		+				

a Strains are abbreviated as follow: *Cpu* = *Claviceps purpurea* 20.1, *Cfu* = *C. fusiformis* PRL 1980, Cpa = *C. paspali* RRC-1481, *Eam* = *Epichloë amarillans* E57, *Ebe* = *E. brachyelytri* E4804, *Eel* = *E. elymi* E56, *Ef*1 = *E. festucae* Fl1, *Ef*2 = *E. festucae* E2368, *Egl* = *E. glyceriae* E2772, *Et*8 = *E. typhina* E8, *Et*5 = *E. typhina* E5819, *Nga* = *N. gansuense* E7080, *Ngi* = *N. gansuense* var. *inebrians* E818, *Nun* = *N. uncinatum* E167, *Pip* = *P. ipomoeae* IasaF13. Symbols: + = present, (+) = intermediate inferred to be synthesized because downstream product is present, − = not predicted and not detected, (−) = predicted but not detected, nt = predicted but not tested, ERA = ergotamine, ERB = ergobalansine, ERC = ergocryptine, ERV = ergovaline. Blank cells indicate compounds not predicted from genotype, and not tested.

b Identification of IDT-436 and terpendoles E, I, J, K, M, M, and A are tentative because authentic standards are unavailable.

Comparisons of ergot alkaloid profiles (Table 4) indicated likely presence, absence, or sequence variation in *EAS* genes among strains (Figure 2). For example, variations in *lpsA* were evident by the production of different ergopeptines, as previously demonstrated for *C. purpurea* [51]. More specifically, grass plants symbiotic with *E. festucae* Fl1 had ergovaline, morning glories symbiotic with *P. ipomoeae* IasaF13 had ergobalansine, and ergots of *C. purpurea* 20.1 had ergotamine and ergocryptine. Other strains lacked ergopeptines. The principal alkaloids in grass plants with *N. gansuense* var. *inebrians* E818 were simpler lysergyl amides, including high levels of ergonovine (EN), low levels of lysergic acid α-hydroxyethylamide (LAH), and intermediate levels of lysergic acid amide ( = ergine), which can result from breakdown of EN, LAH, or both. Morning glories with IasaF13 also had these simple lysergyl amides, which have been reported from *C. paspali* ergots as well [52]. Other strains produced compounds that were intermediates of the lysergic acid pathway; namely, elymoclavine (EC) produced by *C. fusiformis* PRL 1980, and chanoclavine I (CC) produced by *E. elymi* E56.

Each strain that produced indole-diterpenes had a different major pathway end product, although pathway intermediates were typically detected as well (Table 4). Different profiles were likely to be due to different specificities of *idtP* and *idtQ*, and the presence or absence of combinations of *idtF*, *idtK*, *ltmE*, and *ltmJ* (Figure 3) [53]. As apparent pathway end products, grass plants with *E. festucae* Fl1 had lolitrem B, plants with *N. gansuense* E7080 had paxilline, and morning glories with *P. ipomoeae* IasaF13 had terpendoles. Furthermore, *C. paspali* is reported to produce paspalitrem A [54].

Three different profiles of loline alkaloids (Figure 4) were evident among grass plants symbiotic with epichloae (Table 4). Grasses with *E. festucae* E2368 had primarily *N*-formylloline (NFL), but also the *N-*acetylloline (NAL), *N*-methylloline (NML), *N*-acetylnorloline (NANL), and loline. These alkaloids were also produced *in planta* by *N. uncinatum* E167. Plants with *E. amarillans* E57 and *E. glyceriae* E2772 accumulated NANL, and the plant material with *E. brachyelytri* E4804 accumulated 1-acetamidopyrrolizidine (AcAP).

Peramine, production of which is dependent upon the *perA* gene [16], was detected in grass plants symbiotic with *E. festucae* Fl1, but not with *E. festucae* E2368 (Table 4). This alkaloid was also detected in plants with symbiotic *E. amarillans* E57, *E. elymi* E56 and *E. typhina* E8.

### Ergot alkaloid (*EAS*) loci

**Figure 6:**
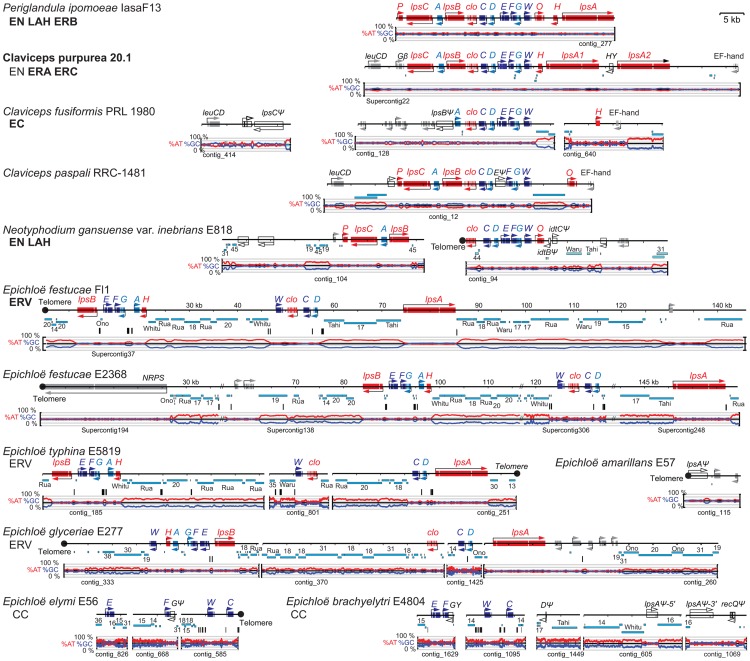
Structures of the ergot alkaloid biosynthesis loci (*EAS*) in sequenced genomes. Tracks from top to bottom of each map represent the following: genes, repeats, MITEs, and graphs of AT (red) and GC (blue) contents. Each gene is represented by one or more boxes representing the coding sequences in exons, and an arrow indicating the direction of transcription. Double-slash marks (//) indicate sequence gaps within scaffolds of the assembled *E. festucae* genome sequences. Closed circles indicate telomeres, and distances from the telomere on the *E. festucae* map are indicated in kilobasepairs (kb). Cyan bars beneath each map represent repeat sequences, and are labeled with names or numbers to indicate relationships between repeats in the different species. Vertical bars beneath the repeat maps indicate MITEs. Gene names are abbreviated *A* through *P* for *easA* through *easP*, *W* for *dmaW*, and *clo* for *cloA*. Genes for synthesis of the ergoline ring system (skeleton) are shown in dark blue for the steps to chanoclavine-I (*W*, *F*, *E*, and *C*), and in light blue (*D*, *A*, and *G*) for steps to agroclavine. Genes for subsequent chemical decorations are shown in red (*clo*, *H*, *O*, *P*, *lpsA*, *lpsB*, and *lpsC*). Identifiable genes flanking the clusters are indicated in gray, and unfilled arrows indicate pseudogenes. The major pathway end-products for each strain are listed below each species name, abbreviated as indicated in Figure 2, and in bold for those confirmed in this study. Note that LAH is a reported product of *C. paspali*, but the sequenced strain is predicted not to synthesize it due to a defective *easE* gene.

In the scaffolded assemblies of the *C. purpurea* and *E. festucae* Fl1 genomes, and the scaffolded E2368 assembly of 2010-06, the *EAS* genes were clustered within individual supercontigs (Figure 6). Also in the assemblies of *C. fusiformis*, *C. paspali* and *P. ipomoeae* genomes functional *EAS* genes were contained in single contigs. Other non-scaffolded assemblies had *EAS* genes in two or three contigs, but only in the case of E818 were the *EAS* genes unequivocally separated in two separate clusters. Long-range physical mapping of the *EAS* genes of E2368 confirmed that they were clustered (Figure S2).

Functions determined to date for enzymes in the ergot alkaloid biosynthetic pathway (Figure 2) [7], [55] were consistent with the presence or absence of specific *EAS* genes (Figure 6) in strains with particular ergot-alkaloid profiles (Table 4 and Table 5). Furthermore, genes without experimentally determined roles in the pathway could be linked with hypothesized steps on the basis of the functions predicted from their sequences, their presence in clusters among strains that produce specific ergot-alkaloid forms, and their absence from fungi lacking those forms. For example, *easH* was predicted to encode a nonheme-iron dioxygenase, and was present in all ergopeptine-producing strains and absent from most ergopeptine nonproducers, suggesting that EasH may catalyze oxidative cyclization of ergopeptams to ergopeptines. Likewise, *easO* and *easP*, were discovered within the *EAS* loci upon sequencing the genomes of the two LAH producers, *P. ipomoeae* and *N. gansuense* var. *inebrians*, and were absent from strains of species not known to produce LAH. These genes were also present in *C. paspali*, but the sequenced strain had a defective *easE*, and for this reason was not predicted to produce ergot alkaloids. Nevertheless, the fact that other *C. paspali* strains are reported to produce LAH [52] strengthens the association of *easO* and *easP* with LAH production.

**Table 5:** Alkaloid biosynthesis genes in sequenced isolates.^a^

		Strain															
Gene	Function	*A* *t* *a*	*C* *f* *u*	*C* *p* *a*	*C* *p* *u*	*E* *a* *m*	*E* *b* *e*	*E* *e* *l*	*E* *f*1	*E* *f*2	*E* *g* *l*	*E* *t*5	*E* *t*8	*N* *g* *a*	*N* *g* *i*	*N* *u* *n*	*P* *i* *p*
***EAS:***																	
*easP*	Hydrolase			+											+		+
*lpsC*	Nonribosomal peptide synthetase		Ψ	+											+		+
*easA*	FMN-containing oxidoreductase		+	+	+				+	+	+	+			+		+
*lpsB*	Nonribosomal peptide synthetase		Ψ	+	+				+	+	+	+			+		+
*cloA*	Cytochrome P450 monooxygenase		+	+	+				+	+	+	+			+		+
*easC*	Catalase		+	+	+		+	+	+	+	+	+			+		+
*easD*	Short-chain alcohol dehydrogenase		+	+	+		Ψ		+	+	+	+			+		+
*easE*	Oxidoreductase		+	Ψ	+		+	+	+	+	+	+			+		+
*easF*	N-Methyltransferase		+	+	+		+	+	+	+	+	+			+		+
*easG*	Reductase		+	+	+		Ψ	Ψ	+	+	+	+			+		+
*dmaW*	Dimethylallyltryptophan synthase		+	+	+		+	+	+	+	+	+			+		+
*easO*	FAD-dependent monooxygenase			+											+		+
*easH*	Nonheme iron dioxygenase		+		+ Ψ				+	+	+	+					+
*lpsA*	Nonribosomal peptide synthetase				++	Ψ	Ψ		+	+	+	+					+
***IDT/LTM:***																	
*idtK*	Cytochrome P450 monooxygenase								+					Ψ			+
*idtM*	FAD-dependent monooxygenase	+		+	+				+					+			+
*idtS*	Unknown	+		+	+				+					+			+
*idtG*	Geranylgeranyl diphosphate synthase	Ψ		+					+					+			+
*idtB*	Unknown	+		+	+				+	+				+			+
*idtC*	Prenyl transferase	+		+	+				+	+				+			+
*idtF*	Prenyl transferase	+		+					+	+				+			+
*idtP*	Cytochrome P450 monooxygenase	+		+	+				+	+				+			+
*idtQ*	Cytochrome P450 monooxygenase	+		+	+				+	+				+			+
*idtE*	Multifunctional prenyltransferase								+								
*idtJ*	Cytochrome P450 monooxygenase								+								
***LOL:***																	
*lolF*	Monooxygenase					+	+			+	+					++	
*lolC*	γ-Class PLP enzyme					+	+			+	+					++	
*lolD*	α-Class PLP enzyme					+	+			+	+			Ψ		++	
*lolO*	Nonheme iron dioxygenase					+	Ψ			+	+			Ψ		++	
*lolA*	Amino acid binding					+	+			+	+			+		++	
*lolU*	Unknown					+	+			+	+			Ψ		++	
*lolP*	Cytochrome P450 monooxygenase					Ψ				+						+Ψ	
*lolT*	α-Class PLP enzyme					+	+			+	+					++	
*lolE*	Nonheme iron dioxygenase					+	+			+	+					++	
*lolN*	Acetamidase									+	Ψ					+	
*lolM*	N-Methyltransferase						Ψ			+						+	
***PER:***																	
*perA*	Multifunctional nonribosomal peptide synthetase, methyltransferase, reductase					+	+	+	+	Δ		Δ	+				

a Abbreviations: *Ata = Aciculosporium take* MAFF 241224, and other strains are abbreviated as in Table 4, except that *Egl* = both *E. glyceriae* strains, E277 and E2772, which had identical sets of alkaloid genes. Symbols are: + = Apparently functional gene, Ψ  = pseudogene, Δ = deleted reductase-encoding domain of *perA* (*perA*-ΔR*). Two symbols in a cell indicate two gene copies.

The genome of *P. ipomoeae* was the only one sequenced that contained functional orthologs of all 14 *EAS* genes (Figure 6), and this was the only strain that produced EN, LAH, and an ergopeptine (ergobalansine). (The *Metarhizium* genomes described in [46] contained all of these genes, but some either had defects or sequencing errors.) Orthologs of twelve of these genes were clustered in the *C. purpurea* 20.1 genome, which had two *lpsA* genes consistent with production of two different ergopeptines (Figure 6). Also, based on gene content, *C. purpurea* 20.1 was predicted to produce EN, though this was not tested. The absence of a functional *lpsB* gene in *C. fusiformis* PRL 1980 accounts for termination of its ergot alkaloid pathway at an earlier position. This strain produces EC, although there was no obvious *EAS* cluster gene for a (mono)oxygenase to catalyze the final step from agroclavine to elymoclavine. The required enzyme seems likely to be encoded either by a non-cluster gene or the *C. fusiformis* isoform of *cloA*. The genome of *N. gansuense* var. *inebrians* E818 lacked only *lpsA* and *easH* in keeping with its chemotype as a producer of EN and LAH, but not of ergopeptines. In contrast, *E. glyceriae* E277, *E. typhina* E5819, and the two *E. festucae* isolates lacked *lpsC*, consistent with the observation that *E. festucae* Fl1 produced an ergopeptine (ergovaline) but not EN or LAH. The fact that no ergot alkaloids were detected in plants with *E. festucae* E2368 reflected the observation that most of the *EAS* genes in E2368 were not expressed (data not shown). Finally, *E. brachyelytri* E4804 and *E. elymi* E56 had functional copies of only the first four pathway genes, which accounted for the observed accumulation of CC in plants symbiotic with E56.

### Indole-diterpene (*IDT*) loci

**Figure 7:**
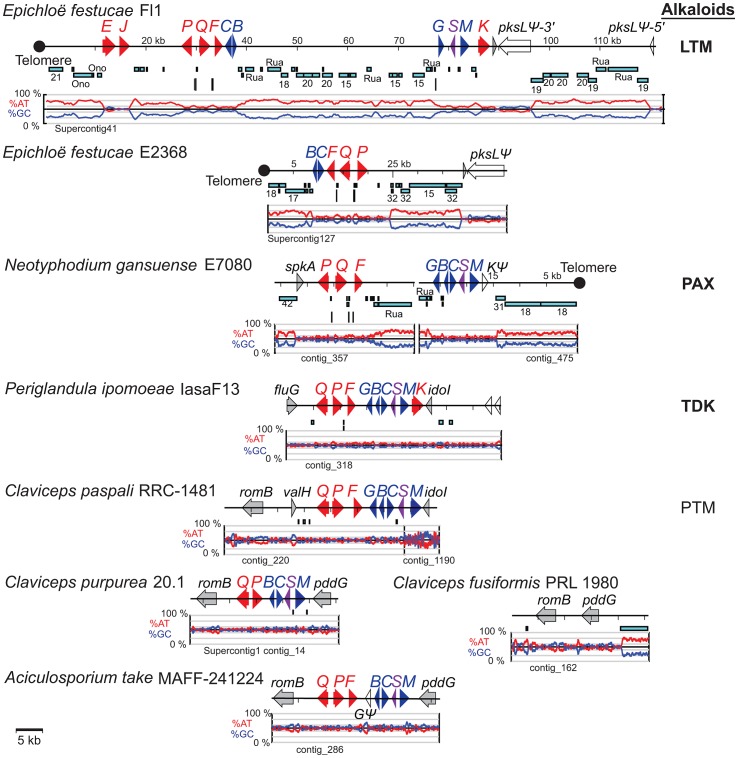
Structures of the indole-diterpene biosynthesis loci (*IDT/LTM*) in sequenced genomes. *IDT/LTM* genes are indicated by single letters, whereby *Q = idtQ* or *ltmQ* (in *E. festucae*), and so forth. Tracks from top to bottom of each map represent the following: genes, repeats, MITEs, and graphs of AT (red) and GC (blue) contents. Each gene is represented by a filled arrow indicating its direction of transcription. Closed circles indicate telomeres, and distances from the telomere on the *E. festucae* map are indicated in kilobasepairs (kb). Cyan bars representing repeat sequences are labeled with names or numbers to indicate relationships between repeats in the different species. Vertical bars beneath the repeat maps indicate MITEs. Genes for the first fully cyclized intermediate, paspaline, are indicated in blue, those for subsequent chemical decorations are shown in red, and *idt/ltmS*, with undetermined function, is in purple. Identifiable genes flanking the clusters are indicated in gray, and unfilled arrows indicate pseudogenes. The major pathway end-product for each strain is listed at the right of its map, abbreviated as indicated in Figure 3, and in bold for those confirmed in this study.

**Figure 8:**
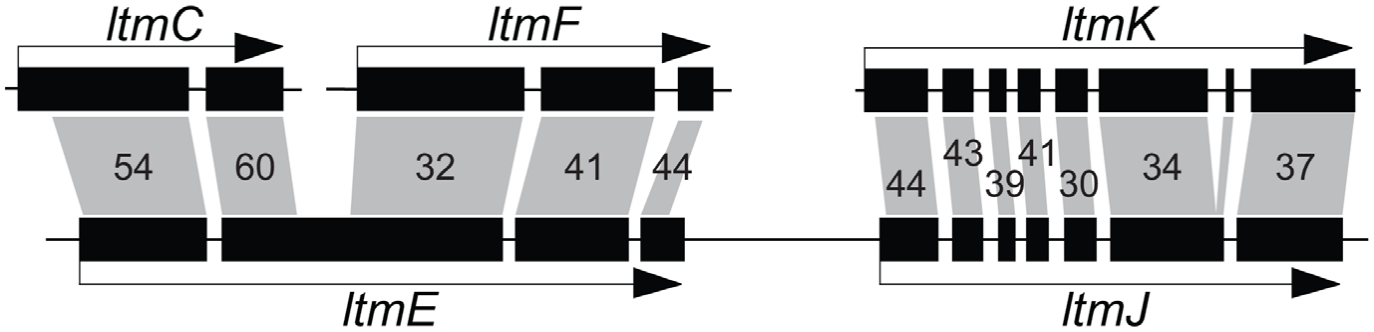
Relationships of *ltmE* and *ltmJ* with other *LTM* genes. Filled boxes indicate coding sequences of exons. Gray polygons indicate closest BLASTp matches to inferred polypeptide sequences for each exon, and are labeled with percent amino-acid identities.

The *IDT* gene clusters in *C. paspali* , *P. ipomoeae*, *N. gansuense* var. *inebrians* and *E. festucae* Fl1 had conserved cores that contained the four genes for synthesis of paspaline (*idt/ltmG*, *M*, *B*, and *C*) (Figure 7). The gene cores also included the newly discovered gene *idt/ltmS* (discussed below) that was conserved in all indole-diterpene producers. Genes *idt/ltmP*, *Q*, *K*, and *F*, which by virtue of their presence, absence or sequence variation determine the particular forms of indole-diterpenes produced [56], were identified in the periphery of each cluster. Two more peripheral genes, *ltmE* and *ltmJ*, were present in the lolitrem producer, *E. festucae* Fl1, but not in the other sequenced genomes (Figure 7, Figure S3). Reciprocal blast analysis of inferred protein products, as well as identification of conserved intron locations, indicated that LtmJ was most closely related to LtmK with 36% overall identity (Figure 8). Furthermore, LtmE was most closely related to LtmC in its N-terminal region and to LtmF in its carboxy-terminal region. These relationships indicated duplications and neofunctionalizations of indole-diterpene modification genes, whereby *ltmJ* was probably derived from a duplication of *ltmK*, and *ltmE* was probably derived from a fusion of duplicated *ltmC* and *ltmF* genes.

The gene arrangements in the *IDT/LTM* loci were conserved in *Claviceps* species, *P. ipomoeae*, and *A. take* MAFF-241224, and varied slightly in *E. festucae* Fl1 and *N. gansuense* E7080 (Figure 7, Figure S3). The gene order in *N. gansuense* E7080 differed by an inversion of the block containing peripheral genes *itdP* and *idtQ*. In turn, the gene order in *E. festucae* Fl1 differed from that of E7080 by an additional inversion of the segment containing three core genes, *idt/ltmC*, *B*, and *G*. Some strains had alterations that eliminated their potential to produce these alkaloids. Specifically, in *C. purpurea* 20.1 and *A. take* the *idtG* gene encoding the first pathway step was either absent or defective. This and several other *IDT* genes were absent from *E. festucae* E2368, and the remaining epichloae (E8, E56, E57, E167, E277, E2772, E818, E4804, and E5819) completely lacked *IDT* genes (Table 5), although E818 had two remnant *IDT* pseudogenes linked to its telomeric *EAS* locus (Figure 6).

The newly discovered *ltmS* gene was identified within the *LTM* cluster of *E. festucae* Fl1 using RNA-seq data of Fl1 and its Δ*sakA* mutant [57] mapped back to the Fl1 genome. The *ltmS* gene followed the same expression pattern as the other *LTM* genes, being significantly down-regulated in the Δ*sakA* mutant. An ortholog of *ltmS* was identified in each *IDT*/*LTM* gene cluster from *C. pupurea*, *C. paspali*, *A. take*, *P. ipomoeae* and *N. gansuense* E7080 (Figure 7). However, homology search (BLASTp) against the nonredundant protein database identified no orthologs in non-clavicipitaceous fungi, and no protein domains were evident in InterPro analysis. Topology prediction tools HMMTOP [58], TMHMM [59] and TopPred [60] indicated that LtmS contains at least four transmembrane domains. The inferred LtmS peptide sequence was compared to the inferred product of the *paxA* gene, which is located in the *P. paxilli* indole-diterpene cluster gene in a similar orientation between the orthologs of *ltmM* (*paxM*) and *ltmG* (*paxG*) [61]. Although sequence similarity was not significant, hydrophobicity plots (data not shown) suggested a shared transmembrane domain structure. Currently, roles for *paxA* and *ltmS* remain to be elucidated, but their shared characteristics, common placement within orthologous *IDT/LTM* and *PAX* clusters, and co-regulation with other *LTM* genes suggested that they may be required for indole-diterpene production.

### Loline alkaloid (*LOL*) loci

**Figure 9:**
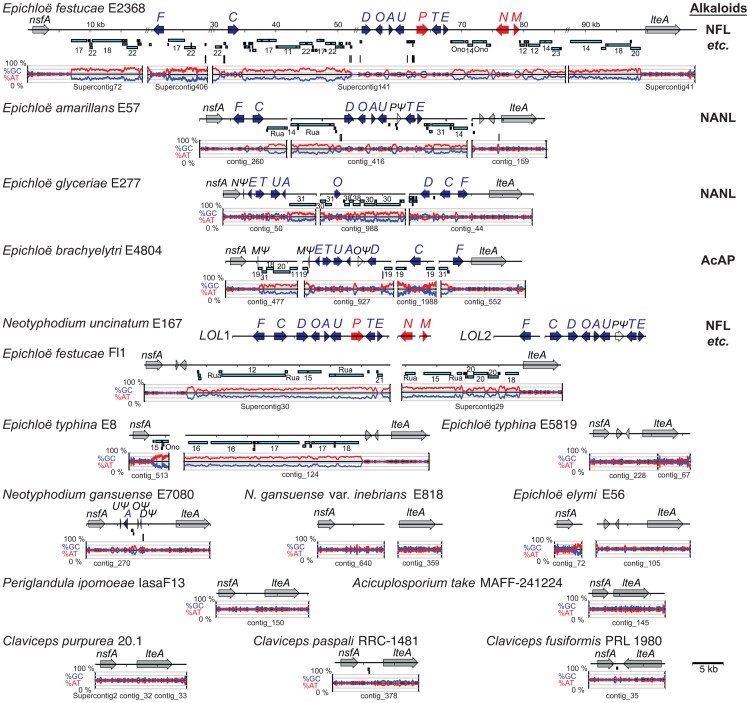
Loline alkaloid biosynthesis loci (*LOL*) in epichloae and the homologous loci in other Clavicipitaceae. *LOL* genes are indicated by single letters, whereby *F = lolF*, *C = lolC*, and so forth. Features are indicated as in Figure 7. Double-slash marks (//) indicate sequence gaps within scaffolds of the assembled *E. festucae* E2368 genome sequence. Genes for the first fully cyclized intermediate, NANL, are indicated in blue, and those for subsequent chemical decorations are shown in red. The major pathway end-product for each strain is listed at the right of its map, abbreviated as indicated in Figure 4, and in bold for those confirmed in this study.

The loline alkaloid biosynthesis (*LOL*) genes were found only in the sequenced genomes of epichloae that produce lolines, and a remnant *LOL* cluster was identified in an additional epichloid strain. Figure 9 compares the *LOL* clusters with the two clusters previously characterized in the hybrid endophyte *Neotyphodium uncinatum* E167 [42]. In the periphery of the *LOL* locus of *E. festucae* E2368 were two divergently transcribed, newly discovered genes designated *lolN* and *lolM*. Orthologous *lolN* and *lolM* genes were also identified in survey sequencing of E167, which has a similar loline alkaloid profile to that of E2368, adding support to the hypothesis that these genes specify certain loline-decorating steps. Scaffolding and long-range physical mapping confirmed and extended previous analysis of large-insert clones [62], indicating that the *LOL* gene order in E2368 was similar to that in E167. In E2368, 10 of the 11 *LOL* genes were in pairs of divergently transcribed genes. In the other strains the precise *LOL*-gene orders were not completely elucidated, but no rearrangements within the cluster were evident. However, orientation of the *LOL* clusters relative to flanking housekeeping genes, *nsfA* and *lteA*, were not conserved. Also, several loline-alkaloid producers had missing or inactive decoration genes (*lolN*, *lolM*, and *lolP*). The *LOL* cluster of *E. brachyelytri* E4804, which accumulates AcAP without an ether bridge, had an inactive *lolO* gene due to an internal deletion, and also lacked functional *lolN*, *lolM*, and *lolP* genes.

No *LOL* genes were identified in *E. typhina* isolate E8 or E5819, *E. festucae* Fl1, *N. gansuense* var. *inebrians* E818, or *E. elymi* E56. Orthologs of the genes that flank *LOL*—namely, *nsfA* and *lteA*—were linked in the E5819 genome, with two additional hypothetical genes between them (Figure 9). The hypothetical genes were also associated with *lteA* in E8, E56, and *E. amarillans* E57, and *nsfA* in Fl1, although the orientation of the genes differed in E57 and Fl1. In genome assemblies of E8, Fl1, E56, and E818 linkage of *nsfA* and *lteA* was not established, and large repeat blocks were identified downstream of *nsfA* and upstream of *lteA* in E8 and Fl1. There was no indication of any *LOL* genes in the genomes of *Claviceps* species, *A. take*, or *P. ipomoeae*, and the *nsfA* and *lteA* orthologs were closely linked in all, although *lteA* was reoriented in *C. fusiformis* PRL 1980 (Figure 9).

### Peramine (*PER*) loci

**Figure 10:**
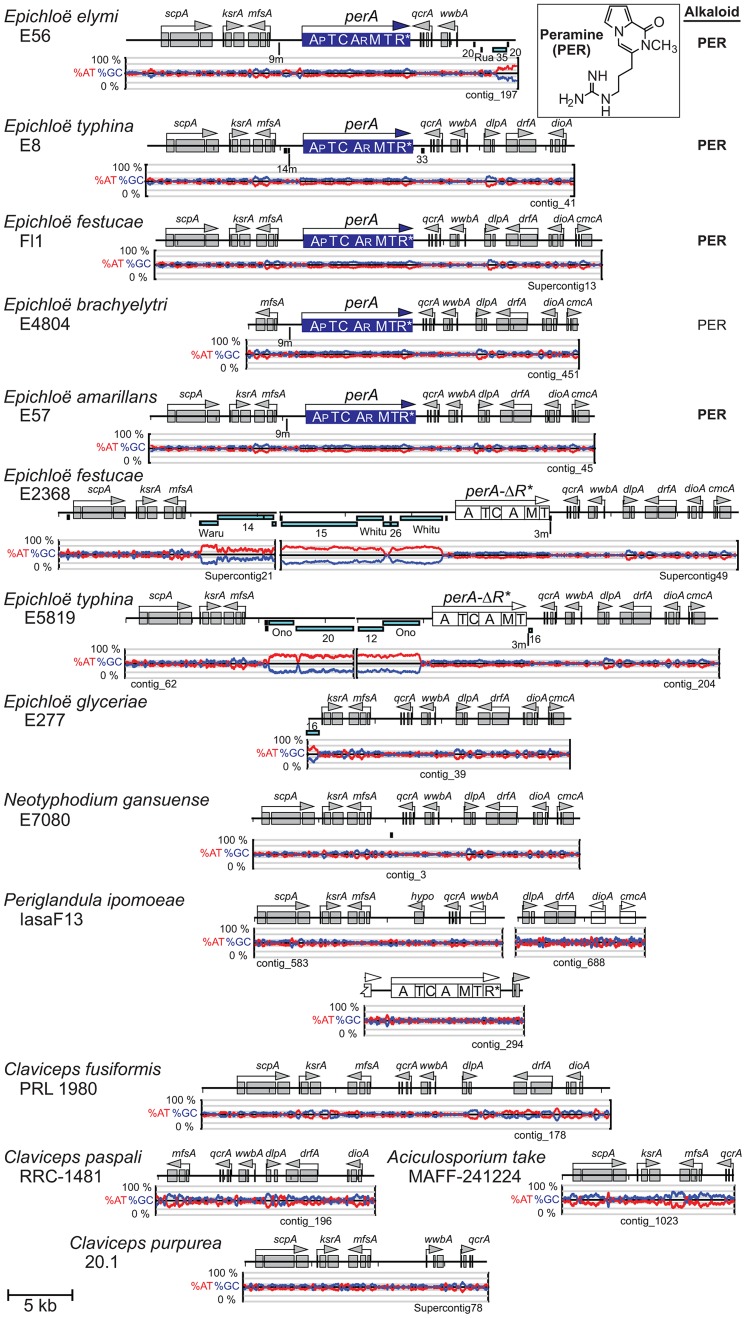
Peramine biosynthesis loci (*PER*) in epichloae and the homologous loci in other Clavicipitaceae. On each map *perA* is color-coded blue for a complete gene and as an open box for *perA-*ΔR*. Domains of *perA* are indicated as A (adenylation), T (thiolation), C (condensation), M (*N*-methylation) and R* (reduction). Subscripts indicate postulated specificity of adenylation domains for 1-pyrroline-5-carboxylate (A_P_) and arginine (A_R_) [16]. Other features are indicated as in Figure 7.

As was the case for the other alkaloid loci, the peramine (*PER*) locus was variable, containing the entire multifunctional *perA* gene in the peramine producers, no *perA* gene, or a partially deleted *perA* gene designated *perA*-ΔR* (Figure 10). The complete gene encodes a multifunctional protein with peptide synthetase, methyltransferase and reductase domains that together may be sufficient for synthesis of peramine [16]. The *PER* locus in *E. festucae* E2368 and *E. typhina* E5819 shared identical deletions of the C-terminal reductase domain. Nevertheless, *perA*-ΔR* was expressed in E2368, raising the possibility that this form also encodes a multifunctional protein, which may participate in the biosynthesis of a metabolite related to peramine if an appropriate thioesterase, condensation, cyclization or reduction domain is provided in *trans*.

Long, syntenous regions flanked both the 5′ and 3′ sides of the functional *perA* genes of *E. typhina* E8, *E. festucae* Fl1 and *E. elymi* E56 as well as the complete and probably functional *perA* genes in *E. brachyelytri* E4804 and *E. amarillans* E57 (Figure 10). The 5′ region included the divergently transcribed gene *mfsA*. The two genomes with *perA*-ΔR* shared synteny of the 3′ flanking region, but repeat blocks in the 5′ flanks apparently disrupted sequence assembly.

A possible *perA* ortholog was identified in *P. ipomoeae*, but it was a pseudogene, and was located in a different locus from the *PER* locus of the epichloae (Figure 10). The predicted gene product included all of the domains of PerA in the same order, with 47.6% amino acid sequence identity over 98.8% of the length of PerA.

### Telomere positions relative to alkaloid loci

In the epichloae, *EAS* and *IDT/LTM* loci were almost always linked to telomeres but *LOL* and *PER* loci were not. In contrast, no telomere linkage of alkaloid loci was evident in *Claviceps* species, *A. take* or *P. ipomoeae*.

Out of the eight epichloae with *EAS* genes, seven had *EAS* clusters linked to terminal telomeres (Figure 6, Figure S2). Long-range mapping of *EAS* genes, telomeres, and other specialized (secondary) metabolism (SM) genes of *E. festucae* E2368 indicated that its *EAS* cluster was linked to a 6-module nonribosomal peptide synthetase (NRPS) gene located near a telomere (Figure S2). Other epichloae had terminal telomere repeat arrays on a contig or supercontig containing some or all of their *EAS* genes. The sole exception was *E. brachyelytri* E4804, which had a RecQ helicase pseudogene near an *lpsA* gene fragment, suggesting possible telomere linkage [63]. Interestingly, although the *EAS* cluster of *N. gansuense* var. *inebrians* E818 was arranged similarly to that of *P. ipomoeae* and the *Claviceps* species, it was broken into two clusters, one of which ended in a telomere located one bp from the *cloA* stop codon. In contrast, the *C. purpurea EAS* cluster clearly was not near a telomere, since it spanned positions 235,054 to 290,780 of the 464,384-bp Supercontig22, and that supercontig had no telomeric repeats at either end.

Among the *IDT/LTM* loci the functional clusters in *N. gansuense* E7080 and *E. festucae* Fl1, as well as the partial cluster in *E. festucae* E2368, were all telomere-linked (Figure 7). (The terminal telomere sequence adjacent to E2368 *LTM* was evident in the 2010-06 assembly, which is also posted at www.endophyte.uky.edu.) Like the *EAS* loci, these *IDT/LTM* loci had the telomeres at different relative positions. (Interestingly, although *N. gansuense* var. *inebrians* E818 lacked functional *IDT* genes, it had two remnant *IDT* pseudogenes adjacent to the telomere-linked *EAS* cluster, as indicated in Figure 6.) In contrast to the epichloid *IDT/LTM* clusters, the orthologous cluster in *C. purpurea* 20.1 was not telomere-linked. This cluster (which was predicted to be nonfunctional because it lacked *idtG*) extended from positions 574,647 to 587,656 on the 978,494-bp Supercontig1 of the *C. purpurea* assembly, and no telomere was present on this scaffold. Also, no telomeres were present on the contigs containing *IDT* genes in the genomes of *A. take*, *C. paspali*, or *P. ipomoeae*.

The *LOL* clusters were not near telomeres. Instead, in all loline-alkaloid producers the clusters were flanked on both sides by groups of housekeeping genes. Published analysis of *E. festucae* large-insert clones [62] indicated that *lolF* is linked to *nsfA*, and *lolT* and *lolE* are linked to *lteA*. The *nsfA* gene was near the end of the 148,125-bp Supercontig71, and the *lteA* gene was near the end of 217,442-bp Supercontig41, and neither of these scaffolds had a telomere end. Likewise, the *PER* loci with complete *perA* genes were not subtelomeric, and no terminal telomere repeats were present on contigs with *perA*-ΔR*.

### Synteny with the *Fusarium graminearum* PH-1 genome

The genome of *F. graminearum* PH-I is almost completely assembled into its known linkage groups [64], and because this species is within the same order (Hypocreales), but a different family from the Clavicipitaceae, we considered it appropriate to compare regions of the alkaloid loci for synteny with the *F. graminearum* genome. None of the four alkaloid loci in the Clavicipitaceae was present in the *F. graminearum* genome. In cases where the alkaloid loci were subtelomeric, flanking genes on their centromeric sides were not orthologous to *F. graminearum* genes. Alkaloid loci that were not subtelomeric and had flanking genes orthologous to *F. graminearum* genes were the *EAS* loci of *Claviceps* species, *IDT* loci of *Claviceps* species and *A. take*, and *LOL* and *PER* loci of the epichloae. The genes flanking the *EAS* clusters of *Claviceps* species were linked and similarly oriented in a syntenous region of the *F. graminearum* genome (Figure S4A). In contrast to the *EAS* loci, the genes flanking *Claviceps* and *A. take IDT* clusters were not syntenous in the *F. graminearum* genome (Figure S4B). The *F. graminearum* orthologs of the *LOL*-flanking genes were contained in a syntenous block (Figure S4C). Likewise, as reported previously [16], *perA* of *E. festucae* Fl1 had apparently been inserted into a block of genes syntenous with their *F. graminearum* orthologs (Figure S4D). These observations raise the possibility that the non-terminal *EAS*, *LOL* and *PER* loci had inserted into their respective genome locations, but where they originally assembled cannot be discerned because no intermediate stages in the evolution of the alkaloid gene clusters have yet been identified.

### Repeat blocks in alkaloid loci

The alkaloid loci of most Clavicipitaceae were associated with repeat DNA derived from transposable elements, which were often stacked and nested extensively into long blocks. The distribution of repeat blocks in alkaloid loci constituted a major and consistent structural difference distinguishing the epichloae from the other Clavicipitaceae. The epichloae typically had long and dynamic repeat blocks predominantly of transposon relics, interspersed throughout their alkaloid loci. This characteristic was not a reflection of overall repeat content of the genomes, considering that epichloae had proportionately less repeat content than *C. fusiformis* and *A. take* (Table 2).

Repeat blocks at alkaloid gene loci were usually very AT-rich. RIP-index analysis (Figure S5) indicated that this was most likely due to the repeat-induced point mutation (RIP) process of selective C to T transitions that is common in fungi [65]. The possibility of RIP was further substantiated by the identification of homologs of the *Neurospora crassa rid-1* gene [66] in all of the sequenced genomes except that of *C. purpurea* 20.1, which paradoxically had one of the lowest repeat contents (Table 2). In most Clavicipitaceae the overall GC content of repeat sequences was very low (Table 3). An exception was *C. purpurea* 20.1, consistent with its lack of *rid-1* homolog and therefore presumed inability to perform RIP. Among the epichloae, the GC contents of *E. glyceriae* repeats tended to be relatively higher, suggesting less history of RIP since the repeat blocks emerged in that lineage.

**Figure 11:**
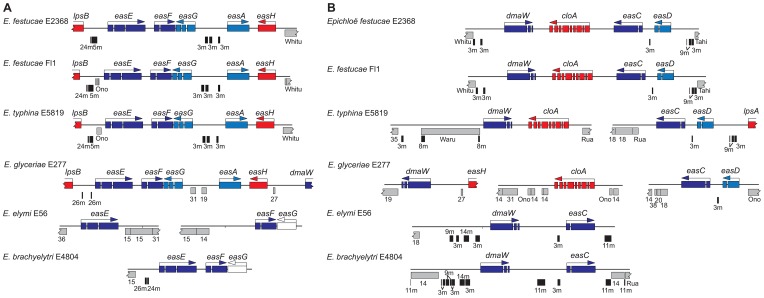
Fine-mapping of repeats in two regions of the *EAS* clusters of epichloae. (A) The *easE-easF-easG* regions. (B) The *dmaW-cloA-easC-easD* regions. Genes are colored as in Figure 6. AT-rich repeats are in gray, and named or numbered to indicate relationships between repeats in the different species. MITEs are indicated by labeled vertical black bars. In some cases, the gene cluster orientation is different from those shown in Figure 6 to facilitate gene alignment. The Waru element is an autonomous parent element of MITE 8m.

The *EAS* and *IDT* clusters of *Claviceps* species, *P. ipomoeae* and *A. take* had very little repeat DNA within them, although repeat blocks flanked the *EAS* clusters of *C. fusiformis* PRL 1980 and *C. paspali* RRC-1481 (Figure 6, Figure 7). These *Claviceps* strains had lost or inactivated one (*C. paspali*) or all (*C. fusiformis*) lysergyl peptide synthetase (*lps*) genes at the cluster periphery, but the conserved-gene cores of their *EAS* and *IDT* clusters remained nearly free of repeat sequences. In contrast, the epichloae all had long blocks of repeat sequences associated with their alkaloid gene loci, and (except for the *EAS* loci of E818, which were divided by a telomere) all *EAS* and *IDT* loci of epichloae were broken up further into subclusters by such long repeat blocks. Even within subclusters a large number of MITE insertions were evident in intergenic regions (Figure 11).

The positions and lengths of repeat blocks and arrangements of MITEs within *EAS* and *IDT* clusters, as well as the gene orders and orientations, widely varied among the sequenced epichloae (Figure 6, Figure 7). Expansions and losses of repeat blocks resulted in variation with respect to the grouping of genes within subclusters. The repeat blocks often extended well beyond the alkaloid gene loci. For example, they dominated the entire Fl1 267-kb scaffold, Supercontig41, extending from the telomere, separating the *LTM* genes into three clusters, and disrupting a polyketide synthase gene in an adjacent SM gene cluster (Figure 7).

In some cases, the order of repeat insertions could be identified within the gene clusters. For example, in *E. festucae* Fl1 the *easD-lpsA* intergenic region apparently had been invaded by Tahi, which in turn was invaded by repeat number 17 (Figure 6). However, many of the multiple repeat insertions were much more complex than this example and varied among the different species. Several differences in MITE positions relative to the genes also appeared to be related to insertions of one or more repeat elements. By example the Waru DNA-transposon relic adjacent to *dmaW* in *E. typhina* E5819 had displaced the proximity of the 3m MITE (Figure 11A), and MITEs adjacent to *easE* in *E. elymi* may have also been displaced by the insertion of a repeat (Figure 11B). Compared to the other epichloae, *E. glyceriae* E277 had fewer MITEs in its *EAS* cluster and throughout its genome.

The *perA* and *LOL* genes were only found in epichloae (Figure 9, Figure 10). Nevertheless, *LOL* loci resembled the other epichloid alkaloid gene clusters in that they contained multiple blocks of nested repeats. Furthermore, positions of the repeat blocks in *LOL* varied greatly between strains, even though the gene orders and orientations appeared to be stable. Repeats in the *PER* loci were associated with *perA*-ΔR* rather than *perA* (Figure 10). In those strains with the deleted R*domain, repeat blocks extended upstream of *mfsA* to the contig ends, leaving it unresolved whether *mfsA* and *perA*-ΔR* were linked. Also, MITE 3m was immediately downstream of the *perA*-ΔR* coding sequence, thus associated with the R*-domain deletion, as previously noted [67].

**Figure 12:**
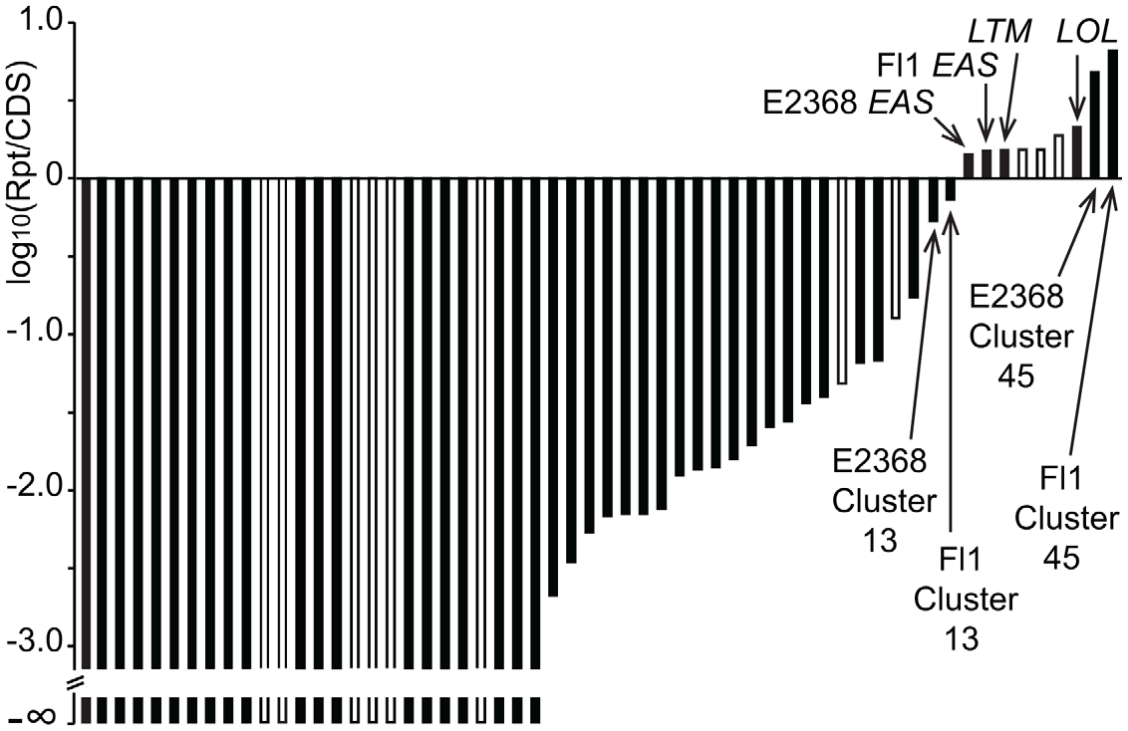
Relative repeat contents in specialized metabolite clusters of *Epichloë festucae*. Log-ratios of repeat sequences (Rpt) to coding sequences (CDS) are shown in order of increasing proportions of repeats. Open boxes represent clusters that are apparently nonfunctional due to inactivation of signature genes.

In order to assess whether large repeat blocks were mainly a feature of epichloid alkaloid clusters or, alternatively, a general feature of their SM clusters, the SM loci of both sequenced *E. festucae* strains as well as *C. purpurea* were manually identified and delineated (Table S4), and the proportions of repeat and coding sequences were determined. Each of these genome assemblies had been scaffolded by paired-end or mate-pair reads. In *C. purpurea* 20.1, repeat sequences within SM clusters were rare and small, though large repeat blocks flanked three SM clusters. For the SM clusters of the *E. festucae* strains, a logarithmic plot of total repeat sequence versus coding sequence lengths (Figure 12) demonstrated that only two active SM loci had comparable proportions of repeat sequence as the *EAS*, *LTM*, and *LOL* loci.

## Discussion

Alkaloids play a major role in the ecology of many Clavicipitaceae, protecting seeds and foliage of host grasses and morning glories from herbivores, or protecting fungal structures (such as ergots) from fungivores. Typically the effects of alkaloids on animals (insects, mammals, etc.) are much more immediate than is the case for many other specialized metabolites because alkaloids target the nervous systems and directly affect behavior [1]. Systemic symbionts such as epichloae and *Periglandula* species supplement the diversity of protective metabolites in grasses and morning glories, respectively, and such diversification should serve an important role in bet-hedging [68], [69] to enhance overall fitness in populations of plant-fungus symbiota on an ecologically variable landscape. Alkaloid diversification occurs at two levels, one being the presence or complete absence of each of several different classes, and the other being variations within each class. Here we compared alkaloid profiles and total alkaloid gene contents among 15 Clavicipitaceae, and also compared the arrangements of those genes and their associations with telomeres and blocks of repeat sequences. Two noteworthy patterns emerged. First, in most alkaloid loci in most species, the periphery of each cluster was enriched in genes that by virtue of their presence, absence, or sequence variations determined the diversity of alkaloids within the respective chemical class. Second, alkaloid gene loci of the epichloae had extraordinarily large and pervasive blocks of AT-rich repeats derived from retroelements, DNA transposons, and MITEs. In the epichloae these gene clusters were clearly unstable, probably because of the repeat blocks and, in the cases of *EAS* and *IDT/LTM* clusters, nearby telomeres. This instability was manifested in strains that had lost complete clusters, strains that had lost large portions of clusters, and strains with variant alkaloids attributable to gene duplications and neofunctionalizations. Partial or complete losses of alkaloid gene clusters generated diversity both between and within species of epichloae, as was apparent in comparisons of two isolates from each of three species, *N. gansuense*, *E. festucae*, and *E. typhina*. Also, gene duplications and neofunctionalizations resulted in the two novel *IDT* genes, *ltmE* and *ltmJ*, required for lolitrem B biosynthesis in *E. festucae*. Here we discuss how the alkaloid locus architectures relate to chemical diversity for each class of alkaloids, and how different ecological contexts of these fungi might select for those architectural differences.

Comprehensive genome sequencing was necessary to identify, with high confidence, all biosynthesis genes for each class of alkaloids in each fungal strain. Every indication has been that, like many fungi, the Clavicipitaceae tend to cluster these genes [7], [42], [70]. However, traditional methods have proven slow and unreliable for complete characterization of each cluster. Cloning and genome walking through these regions is especially difficult when, as is typical of the epichloae, they contain very large blocks of repeat DNA sequence, most of which is highly AT-rich, and cloned fragments containing these sequences are unstable and underrepresented in most gene libraries [42], [43], [71]. Therefore, current genome sequencing technologies facilitated not only a more comprehensive analysis of the gene clusters (including flanking repeats), but also the identification of previously unknown genes in or near these loci. In this way, two genes were newly discovered in the peripheries of some *EAS* loci (*easO* and *easP*), and two new *LOL* genes were also discovered (*lolN* and *lolM*). Furthermore, transcriptome analysis revealed the *ltmS* gene, which eluded *de novo* gene prediction, but was present in all *IDT/LTM* loci of the clavicipitaceous fungi.

Although the role of *ltmS* is not yet apparent, reasonable hypotheses for roles of the newly discovered *EAS* and *LOL* genes could be formulated based on gene presence or absence, along with comparisons of alkaloid profiles. For example, *easO* and *easP* were associated with LAH production. The sequence of *easO* indicates that it encodes a flavin-binding monooxygenase that, in the context of LAH biosynthesis, may oxidize the α-carbon of the alanine-derived residue in ergonovine or the ergonovine precursor attached to the LpsB/LpsC peptide synthetase complex. Furthermore, the sequence of *easP* indicates that it encodes an α/β hydrolase-fold protein, which could be involved in subsequent hydrolysis to release LAH. Similarly, the presence of *lolN* and *lolM*, predicted to encode an acetamidase and an *N*-methyltransferase, respectively, fits well with late enzymatic steps needed for NFL biosynthesis. Evidence from the genomes and chemotypes of strains with different loline alkaloid profiles suggest that the first fully cyclized loline alkaloid is NANL, which has an acetylated 1-amine. In order to produce NFL from NANL, it would be necessary first to deacetylate, then di-methylate that amine to generate NML. These are the likely roles for LolN and LolM, respectively, and the previously characterized *lolP* gene encodes a cytochrome P450 involved in the final process of oxygenating NML to NFL [72].

The Clavicipitaceae are best known for their ergot alkaloids, and among species and strains there are dramatic differences in ergot-alkaloid profiles [2], [73]. This variation is due to the particular mid-pathway or late-pathway genes that they possess, as well as differences in substrate or product specificity due to gene sequence variations [51], [70], [74]. In this study we associated chemotypes of *Claviceps* species with presence or absence of the genes *lpsA*, *lpsB*, *lpsC*, *easH*, *easO* and *easP*. In the *Claviceps* and *Periglandula* species, these genes are all in the cluster periphery, in contrast to the early-pathway and most mid-pathway genes in the core. (The mid-pathway gene *easA* is an exception, but with a unique role in alkaloid diversification as discussed below.) Even in the extensively rearranged *EAS* clusters of the epichloae the *lps* genes are often in the periphery. This placement is interesting considering the key role of *lps* genes in much of ergot-alkaloid diversity [51], and the propensity we observed for transposon-derived repeats to flank the *EAS* clusters in most Clavicipitaceae. Indeed, long repeat blocks were generally evident whenever *lps* genes were partially or wholly deleted or inactivated by extensive mutation.

In addition to gene presence or absence, sequence variation of certain genes resulted in further diversification of ergot alkaloids. This was dramatically evident for the multi-module lysergyl peptide synthase subunit I encoded by *lpsA*. Variations in *lpsA* among genera, and even between the two copies found in *C. purpurea* 20.1 [51], dictate which three amino acids are added to lysergic acid, hence which of 19 known ergopeptines are produced [7]. In addition, *easA*, which encodes a mid-pathway step in synthesis of the first fully cyclized ergoline, is also one for which sequence variation results in diversification of ergot alkaloids. Different *easA* forms determine if ergolines or dihydroergolines are produced [74]. (None of the strains sequenced in this study produce dihydroergolines.) Conceivably, variation in *cloA* also plays a role in ergot-alkaloid diversity. The *C. purpurea* CloA cytochrome-P450 catalyzes oxygenation of elymoclavine to paspalic acid [75], which spontaneously rearranges to lysergic acid. The *cloA* gene from *C. fusiformis* PRL 1980, though expressed and without any apparent defect, fails to complement this role in a *cloA*-deleted strain of *C. purpurea* [70]. However, it is unknown whether the variant form of CloA in *C. purpurea* has another role, for example in the oxygenation of agroclavine to elymoclavine.

Clearly the *EAS* loci in the epichloae are unstable and subject to rearrangements and partial or complete elimination. We characterized genomes of several epichloae that have the 11 genes required to synthesize the complex ergopeptines, others that had only the four functional genes required for chanoclavine I biosynthesis, and still others that lacked any functional *EAS* genes. Extensive rearrangements of the epichloid *EAS* clusters contrasted with the gene arrangements conserved among *Claviceps* species, *P. ipomoeae*, and the published *Metarhizium* genomes [46]. Interestingly, although *N. gansuense* var. *inebrians* E818 had an *EAS* locus structure and chemotype more similar to that of *P. ipomoeae* IasaF13 than to other epichloae, the E818 *EAS* locus had been broken up with a telomere and had lost *lpsA* and *easH*. Therefore, a tendency for rearrangements and telomere associations was consistently evident in, and contributed to, the chemotypic diversity of the epichloae.

The organization of *IDT/LTM* genes showed an even more distinct and consistent positioning of early and late pathway genes compared to the *EAS* loci. Furthermore, sequence variations in essentially all of the peripheral *IDT/LTM* genes account for differences in specificities of the cytochromes P450 and prenyltransferases that they encode, resulting in broad diversity of alkaloids within this class [53]. Rearrangements of *IDT/LTM* genes in the epichloae associated with large repeat blocks and telomeres were probably also responsible for gene duplications and neofunctionalizations that generated two new peripheral genes (*ltmE* and *ltmJ*) in the *LTM* cluster, allowing *E. festucae* to produce an especially complex group of indole-diterpenes, the lolitrems. This is a dramatic illustration of chemical diversification by cluster rearrangements almost certainly facilitated by the blocks of transposon-derived repeats.

The *LOL* loci, which were found only in epichloae, had features similar to *EAS* and *IDT/LTM*. Two of the three decoration genes identified in *E. festucae* E2368 were at the locus periphery, and all functional *LOL* loci were riddled with large and dynamic blocks of transposon-derived repeats. One notable difference was that, unlike the *EAS* and *IDT/LTM* clusters, the *LOL* clusters were not subtelomeric. Nevertheless, like *EAS* and *IDT/LTM*, the *LOL* loci were subject to partial or complete loss, resulting in different loline alkaloid profiles. Even the *PER* locus of some strains contained repeat blocks, and the *perA* gene also exhibited instability.

It is noteworthy that ergot alkaloids and indole-diterpenes are known among diverse ascomycetes, for which they undoubtedly have a variety of ecological roles. For example, the presence of *EAS* and *IDT* genes in *Metarhizium* species could indicate that these neurotropic alkaloids contribute to their abilities to affect behavior of parasitized insects. In contrast, peramine and loline alkaloids are characteristic of the epichloae, but unknown among other fungi. Consequently, compared to other Clavicipitaceae the epichloae have an even more diverse pallet of alkaloids to draw on to protect host plants. As systemic, and often vertically transmitted symbionts, epichloae depend on host plants throughout their life cycles, so it is to be expected that such an arsenal of plant protectants greatly benefits the epichloae.

The dynamics of alkaloid loci in the Clavicipitaceae, and especially the epichloae, promote chemotypic diversification even within species, and with respect both to the classes of alkaloids as well as the particular structures within each class that are produced. Transposon-derived repeats such as typify these loci can promote both recombination and mutation, and their insertions or deletions can radically alter gene regulation [76], [77]. We suggest that selection for chemotypic diversification within epichloid species may be imposed by their exceptional variety of life histories and host interactions. Whereas most other Clavicipitaceae are either contagious parasites (*A. take* and *Claviceps* species) or vertically transmitted mutualists (*P. ipomoeae*), epichloae vary widely in relative mutualistic or parasitic effects on their hosts based largely on transmission mode, and many (e.g., *E. amarillans*, *E. brachyelytri*, *E. elymi*, *E. festucae* and, in some hosts, *E. typhina*) have the remarkable capability to exhibit both transmission modes simultaneously on different tillers of the same plant [25]. Variation in relative vertical or horizontal transmission is expected to impose variation in selection on the symbiont, whereby vertical transmission selects for enhancements of host fitness [78]. Alkaloids, which typically deter herbivores [1], can be major contributors to host fitness, but also expensive to produce [79]. We suggest that variation in life history traits among the epichloae, as well as variation in ecological settings of their hosts, selects for exceptionally dynamic alkaloid loci that ensure high interspecific and intraspecific chemotypic variability.

## Materials and Methods

### Biological materials

Fungal strains and their sources are listed in Table S1. The *Epichloë* and *Neotyphodium* species, *Claviceps fusiformis*, *Claviceps paspali*, and *Aciculosporium take* were cultured on potato dextrose agar (PDA) on a cellophane layer, or in potato dextrose broth (PDB) with shaking at 23°C. Mycelia were collected by centrifugation for 20 min at 5525× *g*, frozen and lyophilized prior to DNA isolation. Culture conditions for *C. purpurea* were as in Mey et al. [80].

Because *Periglandula ipomoeae* is so far nonculturable, the adaxial sides of the leaves of an infected host plant (*Ipomoea asarifolia*) were wetted with deionized water, and mycelia were picked off with a scalpel, placed into a vial with 70% ethanol, and stored at −20°C. The mycelium was harvested by centrifugation, frozen and lyophilized.

DNA was isolated by the method of Al-Samarrai et al. [81] or, for *C. purpurea*, by the method of Cenis [82].

### Microscopic examination of *Epichloë festucae in symbio*

In order to document the stages of the life cycle of *Epichloë festucae* Fl1, the fungus was transformed with the plasmid, pCA49, which includes an enhanced cyan fluorescent protein (eCFP) coding sequence controlled by the *Pyrenophora tritici-repentis TOXA* gene promoter [83]. Fungal transformation was performed as previously described [72] and transformants were selected for resistance to hygromycin B. The transformants were introduced into seedlings of perennial ryegrass (*Lolium perenne*) [84], and the symbiotic fungus was detected by tissue-print immunoblot with antiserum raised against a protein extract from *Neotyphodium coenophialum* [85]. Plants were grown in the greenhouse, and vernalized to induce flowering and seed development [86]. Plant tissues were dissected manually with the aid of a dissecting scope, placed on a glass slide in a drop of 50% glycerol, and covered with a coverslip. Confocal micrographs were generated with an Olympus FV1000 point-scanning/point-detection laser scanning confocal microscope, equipped with a 440 nm laser. Emission fluorescence was captured and collected at 467±15 nm through the eCFP filter. Image acquisition was performed at a resolution of 512×512 pixels and a scan rate of 20 µs pixel^−1^. The objective, Olympus water immersion PLAN APO 20×-Water (NA 0.75), was used for observing and generating micrographs. FLUOVIEW 1.5 software (Olympus) was used to control the microscope and export images as TIFF files.

### Alkaloid analyses

Methods of analyses were as described previously for ergot alkaloids [75], [87], lolines [88], peramine [89], and indole-diterpenes [90].

### Clone libraries

For Sanger sequencing of the *E. festucae* E2368 genome, a clone library of randomly sheared genomic DNA was constructed as follows. Nuclear DNA was enriched by bisbenzimide-CsCl isopycnic ultracentrifugation, randomly sheared with a GeneMachines Hydroshear (DigiLab Genomic Solutions, Inc.), twice gel-fractionated to select DNA fragments of 3.5–4.5 kb, and cloned into pBCKS+ (Stratagene Cloning Systems, La Jolla, CA, USA). The library consisted of approx. 5 million clones, of which 2.5 million cfu were stored at −80°C as aliquots of transformed T1-phage resistant *Escherichia coli* cells (Electromax DH10B; Invitrogen Corp., Carlsbad, CA, USA), and the remainder as ligation mixture. The *E. coli* transformants were grown on LB agar with chloramphenicol (25 mg/L). Colonies were picked by a QPix robot (Genetix, Hampshire, UK) into 96-deep-well plates with 2× YT medium (1.5 ml per well), and grown overnight in a HiGro (GeneMachines, San Carlos, CA, USA) oxygenated shaking incubator for microtiter plates. The plasmids were purified robotically (Biomek FX, Beckman Coulter Inc, Fullerton, CA, USA) with the Perfect-Prep Plasmid 96 kit (Eppendorf AG, Hamburg Germany). Sequence reactions and capillary electrophoresis were conducted using vector primers and BigDye3.1 (Applied Biosystems, Foster City, CA, USA) at 1/16th reaction strength. The reactions were cleaned by ethanol precipitation and capillary electrophoresis was performed in a model 3730 DNA analyzer (Applied Biosystems). Both ends of each plasmid were sequenced. Sequencing results indicated that 99.8 of the clones contained genomic DNA inserts.

For the *E. festucae* Fl1 genome, a library was prepared in the fosmid vector pCC1FOS (Epicentre). DNA was fragmented with a Hydroshear equipped with the LARGE assembly, at speed setting 36, for 15 cycles. The fragments were end-repaired with the End-It kit (Epicentre), size-selected by electrophoresis in 0.4% agarose gel with Gelgreen stain (Biotium), imaged with blue light, purified from the agarose with Gelase (Epicentre), and blunt-end ligated to the fosmid arms using Fast-link (Epicentre). *Escherichia coli* Epi-300 T1^R^ cells were transformed and selected for chloramphenicol resistance.

### Genome sequencing

All DNA sequencing was conducted at the University of Kentucky Advanced Genetic Technologies Center. Most sequencing was conducted on a Roche/454 Titanium pyrosequencer. DNA was nebulized and size-selected to approximately 600 bp with AMPure beads (Agencourt), and subjected to shotgun pyrosequencing using the GS FLX Titanium General Library Preparation Kit, GS FLX Titanium LV emPCR Kit (Lib-L), and GS FLX Titanium Sequencing Kit XLR70 (Roche). Paired-end pyrosequencing was also conducted for *E. festucae* Fl1 (2-kb fragments 960,278 true paired end reads), *E. festucae* E2368 (3.0-kb fragments, 113,208 true paired end reads), and *Claviceps purpurea* (3.0-kb fragments, 1,128,137 true paired end reads). For paired ends, DNA was sheared with a Hydroshear with standard assembly, 20 cycles at speed setting of 12, then size selected with AMPure beads (Agencourt). The GS FLX Titanium Paired End adaptor set from Roche was used with *Cre* Recombinase, Exonuclease 1, and *Bst* polymerase from NEB according to the Roche GS FLX Titanium 3 kb Paired End Library Preparation Method Manual. Survey sequencing was conducted on and Ion Torrent PGM (Life Technologies) according to manufacturer's instructions. Sanger sequencing of the *E. festucae* E2368 genome was conducted on paired-ends of a library of cloned 3.8-kb (ave.) DNA fragments as described above (119,114 reads incorporated). In addition, the E2368 sequence assembly incorporated 235 reads of ca. 11-kb clones of genomic DNA in the pJAZZ system (Lucigen), as well as directly cloned telomere-containing fragments [91]. Sanger sequencing of the *E. festucae* Fl1 genome was conducted on paired-ends of the fosmid library of cloned 36-kb (ave.) DNA fragments (7259 read-pairs incorporated).

Data sets consisted of 2.3 M to 6.0 M reads per genome. Assemblies of *E. festucae* Fl1 and E2368 genomes incorporated paired-end data from Sanger sequencing in addition to 454 paired-end and single-end pyrosequencing data from a Roche/454 Titanium sequencer. The *C. purpurea* assembly used both single-end and paired-end pyrosequencing reads. All other genome assemblies used single-end pyrosequencing reads, some supplemented with sequences obtained on an Ion Torrent PGM (Life Technologies). Pyrosequencing reads that were duplicates, very short (<80 nt), or very long (>650 nt), or that had more than 1% of uncalled bases, were purged using utility program prinseq-lite-1.5 (http://prinseq.sourceforge.net) as suggested in Huse et al. [92]. Ion Torrent reads were trimmed of all base-calls after the first 230 bases. All genomes were assembled using Newbler Assembler ver. 2.5.3 (Roche/4540) with default parameters and the -sio option to ensure proper order of input data, with single-end reads preceding any paired-end data, and paired-end read libraries (Sanger and pyrosequencing) ordered by increasing insert size. Assemblies were uploaded to GenBank (Table S2), and are provided with annotations on GBrowse web sites (www.endophyte.uky.edu). The annotated assembly of the *C. purpurea* genome sequence can be viewed at http://www.ebi.ac.uk/ena/data/view/Project:76493.

### Annotation of repetitive DNA elements

Repetitive DNA families in the genome sequences were defined by processing a self-BLASTN report from each genome using a custom PERL script (Amyotte S.G. et.al *Manuscript in preparation*) that identified sequences with multiple genome copies and classified these repeats into non-redundant families. The repeat families were then manually curated to correct or remove families misidentified in the automated process above. The genome distribution of repeated sequences was characterized using RepeatMasker version 3.2.9 [93], with Cross_Match [94] version 0.990329, with the final set of repetitive families serving as a custom library. Results have been included in the GBrowse web sites (www.endophyte.uky.edu). All unique repeats identified from the genome custom libraries were compared by reciprocal BLASTn to identify conserved sequences within and between each species. Repeat sequences with BLAST scores greater than 100 were used to develop a matrix table of corresponding repeat numbers. A common number was given to each repeat association to rapidly identify repeat families conserved across species. The matrix table was used to label repeats within each gene cluster with the universal repeat numbers (Figure 6, Figure 7, Figure 9, Figure 10, Figure 11). Repeats were assigned to putative superfamilies, and families where possible, based on BLASTx analysis and the presence and orientation of terminal repeats (Table S5).

Miniature inverted repeat transposable elements (MITEs) previously characterized in *E. festucae* [67] were identified in other genomes by BLASTn using a personal database. To determine whether short repeats found in non-epichloid clusters were MITEs they were analyzed for terminal inverted repeats using einverted (EMBOSS; http://emboss.bioinformatics.nl/cgi-bin/emboss/einverted). Individual repeat-containing loci were aligned using MUSCLE and manually analyzed for evidence of recombination.

### RIP-index analysis

RIP indices were calculated using a sliding window analysis with a 200 bp window and a step size of 20-bp (in the centromere-to-telomere direction). RIP indices (ApT/TpA) [95] were calculated for each window. The process was repeated until the window met the end of the sequence (i.e partial windows were not counted). These operations were performed automatically using a perl script (Protocol S1).

### Gene identification and orthology and phylogenetic analyses

Gene predictions were conducted by various methods available in MAKER version 2.0.3 [96]. In the MAKER runs, assembled contigs were filtered against RepBase [97] model organism “fungi,” using RepeatMasker [93] version open-3.2.8. Our MAKER runs used the predictors AUGUSTUS 2.3.1 (*Fusarium* model) [98], FGENESH 3.1.1 (*Fusarium* model) [99], GeneMark-ES 2.3a (self-trained), and SNAP 2006-07-28 (trained with *C. purpurea* gene predictions for genus *Claviceps*, and with *E. festucae* E2368 gene predictions for other genera). These *ab initio* predictions were supplemented with evidence from Clavicipitaceae proteins in the NCBI non-redundant protein database and from assembled E. festucae ESTs (unigenes). Relationships of predicted proteins to known protein families were assessed by running InterProScan [100] on the inferred protein sequences inferred from the predicted genes. Results, including MAKER and FGENESH predictions and subsequent analyses, have been included in a collection of web sites based on GBrowse 1.70 [101], [102], posted at www.endophyte.uky.edu.

Gene modeling for *C. purpurea* was done similarly, by applying three different gene prediction programs: 1) FGENESH [99] with different matrices (trained with *Aspergillus nidulans*, *Neurospora crassa* and a mixed matrix based on different species); 2) GeneMark-ES [103] and 3) AUGUSTUS [98] with available ESTs as hints. The different gene structures were displayed in GBrowse [101], [102], allowing manual validation of all coding sequences (CDSs). Annotation was aided by BLASTx hits between the *C. purpurea* genome and those from *Blumeria graminis*, *Neurospora crassa*, *Fusarium graminearum* and *Ustilago maydis*, respectively. For the cluster regions and selected genes of interest the best fitting model per locus was selected manually and gene structures were adjusted by manually splitting them or redefining exon-intron boundaries based on EST data where necessary.

Orthology analysis was conducted on FGENESH-predicted proteins with length 10 amino acids or greater. Each inferred protein sequence was assigned a unique label with a prefix indicating its source genome. The predicted genes were first compared to the curated ortholog groups in OrthoMCL-DB [104] version 4 using the OrthoMCL web service (http://orthomcl.org/cgi-bin/OrthoMclWeb.cgi?rm=proteomeUploadForm), to which each predicted proteome was submitted independently. Next, the combined set of inferred proteins from all of the sequenced Clavicipitaceae was analyzed as described in the OrthoMCL algorithm document (http://docs.google.com/Doc?id=dd996jxg_1gsqsp6). The software versions used for this procedure were: OrthoMCL version 2.0.2 [105], MCL-bio [100], MCL version 10–201 (http://micans.org/mcl/) [106], and NCBI BLAST version 2.2.25 [107].

As noted by Li et al. [105], an OrthoMCL-derived “ortholog group” may contain paralogs as well as orthologs. We used the COCO-CL [108] software distribution to recursively divide the ortholog groups obtained from OrthoMCL into sub-groups. A division was accepted if it had a bootstrap score of 0.75 or greater and a split-score (number of taxa common to both sub-groups, divided by the number of taxa represented in the smaller sub-group) of 0.5 or greater; a high split score indicates that the group is likely to be the result of an ancient duplication event, as many taxa have representative protein sequences on both sides of the split. For these analyses, COCO-CL was slightly modified as follows. The multiple amino-acid-sequence alignments employed MUSCLE version 3.8.31 [106] instead of ClustalW (version 1.83) [109] because rigorous experiments with simulated protein data sets, have shown that MUSCLE is comparable or superior in speed and average accuracy to the best current methods, such as CLUSTALW, MAFFT, and T-Coffee [106]. ClustalW was still used to compute distance matrices from MUSCLE's alignments. The remaining calls to ClustalW in the COCO-CL source code were converted to non-interactive mode, avoiding freezes that can occur when ClustalW prompts the user unexpectedly. Finally, we addressed a potential infinite loop generated by the COCO-CL clustering program when a cluster cannot be partitioned into precisely two subclusters. In our version this situation terminates the clustering program, leaving the cluster unpartitioned.

Results of OrthoMCL and COCO-CL have been included in the genome browser web sites (www.endophyte.uky.edu). Clicking an FGENESH prediction in the browser opens a data page that lists and hotlinks the prediction's homologs and orthologs, as well as a link to download the multiple sequence alignment for that cluster. A patch file is provided in Supporting Information (Protocol S2).

For phylogenetic analysis, the following steps were performed on the Phylogeny.fr site [110]: Sequences were aligned with MUSCLE [111], the phylogenetic tree was inferred with PhyML [112], and branch support was estimated by the approximate likelihood-ratio test [113] with the SH-like option. Trees were compared by the Shimodaira-Hasegawa test [114] implemented in phangorn [115] with the default parameters and 10,000 bootstrap replications.

### mRNA sequence analysis

Sequences of cDNA generated by reverse transcription of mRNA provided the information required for manual annotation to refine models of alkaloid biosynthesis genes. Using the Qiagen RNeasy Plant Mini kit, RNA was isolated from symbiota composed of *Lolium pratense* ( = *Schedonorus pratensis*) with *E. festucae* (see Figure 1D). Tissues analyzed were newly emerged stromata and pre-anthesis inflorescences. RNA quality and quantity was checked on Bioanalyzer 2100 (Agilent) using plant RNA nano chip. A clone library was constructed by cDNA synthesis with the SMART kit (ClonTech) [116], normalization, and cloning into the λTriplex2 vector (ClonTech). Transfected *E. coli* BM25.8 cells were grown with ampicillin to select clones, and plasmid DNA was isolated and sequenced by standard Sanger sequencing using BigDye version 3.0 and an Applied Biosystems (Life Technologies) model 3730 xl DNA sequencer.

For deep cDNA sequencing (RNA-seq), 10 µg of high quality total RNA was used for cDNA library preparation according to the mRNA sequencing sample preparation guide (Illumina, Cat# RS 930-1001). Sources of RNA were inflorescences and stromata of *L. pratense-E. festucae* symbiota, laboratory id numbers 2194 and 2352. The libraries were validated on a 2100 Bioanalyzer using a DNA-1000 chip (Agilent). These libraries were used for bridge-PCR (SR cluster generation kit v4, Illumina) and 82-cycle single-read sequencing was conducted on a Genome Analyzer IIx (Illumina) in the DNA Facility at Iowa State University.

RNA-seq data from stromata and inflorescences, as well as RNA-seq data previously obtained from wild type *E. festucae* and a *sak*A mutant [57] were used for genome-wide identification and annotation of the expressed protein-coding genes of the *Epichloë festucae* strains. The RNA-seq data were combined with previously generated RNA-seq data and assembled into TAC contigs (defined as a continuous exonic region with contiguous read coverage) on both E2368 and Fl1 assemblies by MapSplice [117], which performs both spliced and unspliced alignment of RNA-seq reads to the reference genome. Then the combined read alignment coverage of the 6 tissues was used to detect exons. Exon boundaries are determined by splice junctions or the absence of the read coverage. Two TACs can be merged together by a splice junction connecting them. A TAC-contig is a maximum set of TACs that are linked together by splice junctions. If alternative splicing events exist, the alternative splice junction with more read alignment support is preferred. Because intergenic transcription or overlapping transcription of convergent genes sometimes led to merged gene models, junctions that crossed two FGENESH genes were filtered out, and TAC contigs that overlapped with more than two FGENESH-predicted genes were split according to the predicted gene boundaries. The 5′ and 3′ boundaries of gene structures were also trimmed based on the predicted genes.

### Manual gene annotation

All of the genes for ergoline, peramine, indole-diterpene and loline alkaloid biosynthesis, as well as genes used for phylogenetic analysis, were manually annotated. Many of the genes were previously characterized in the same or related species and strains by targeted reverse transcription of their mRNAs followed by cDNA sequencing [21], [39], [41]–[43], [70], [71], [118]–[121]. Transcriptome information from *E. festucae*, including reads from cloned cDNAs and assembled TAC contigs, was used to model the gene exons. Cross-species comparisons, for example by using tBLASTn or tBLASTx, were employed to refine models in species for which transcript data were unavailable. In some cases, such as the newly discovered *easO* and *easP* genes, mRNA segments were amplified by reverse-transcription-PCR, and sequenced.

### Identification and delimitation of specialized metabolism (SM) gene clusters

BLASTx and InterproScan [100] were employed to identify genes encoding enzymes that are signatures of SM gene clusters in the Ascomycota; namely, nonribosomal peptide synthetases (NRPS; IPR010071, IPR006163, IPR001242), polyketide synthases (PKS; IPR013968), DMATS-family aromatic prenyltransferases (IPR017795, Pfam PF11991), and terpene synthases/cyclases (IPR008949). The probable functions of proteins encoded by nearby genes were similarly assessed, as SM gene clusters contain various families of biosynthetic genes including mono- and dioxygenases, dehydrogenases, reductases, pyridoxal-phosphate (PLP)-cofactor enzymes, hydrolases, prenyltransferases and methyltransferases, as well as ABC or MFS efflux pumps and transcription regulators. However, many members of these enzyme families are involved in primary metabolism. Considering that most Clavicipitaceae can grow on minimal salts medium with sugars and inorganic nitrogen, those genes that had orthologs (identified by COCO-CL) among all of the sequenced genomes were considered probable primary metabolism genes. This interpretation was validated by the observation that most apparently active SM signature genes were flanked on one or both sides by ortholog groups with limited distribution among the 12 sequenced genomes. (Note that even after COCO-CL analysis, NRPS and PKS ortholog groups usually had several members in each genome, making it difficult to discern the distribution of their true orthologs, but this was not generally a problem for nearby genes.).

### Accession numbers

Genome and sequence accession numbers are listed in Table S2.

## Supporting Information

Figure S1 Phylogenies of housekeeping genes from sequenced isolates and other Clavicipitaceae. (A) Phylogenetic tree based on nucleotide alignment for a portion of the RNA polymerase II second-largest subunit gene, *rpbB*. (B) Phylogenetic tree based on nucleotide alignment for a portion of the translation elongation factor 1-α gene, *tefA*. Trees are rooted with *Fusarium graminearum* as the outgroup. Epichloae are indicated in green, *Claviceps* species are indicated in blue, *Periglandula* species are indicated in red, and *Aciculosporium take* is in black. Species for which genomes were sequenced in this study are shown in bold type, and asterisks indicate plant-associated fungi.
(EPS)

Figure S2 Physical mapping of *EAS* genes in *Epichloë festucae* E2368. Genomic DNA was digested with *Sfi*I or *Not*I as indicated under each panel, separated by clamped homogeneous electric field (CHEF) electrophoresis, and blotted onto nylon filters. The filters were cut into strips, which were probed with labeled segments of the genes indicated above each lane. Low Range PFG marker (NEBiolabs), used as size standard. (A) DNA digested with *Sfi*I was hybridized to *lpsA* and *lpsB* probes. The result confirmed that the two genes are present on the same *Sfi*I fragment. (B) DNA digested with *Not*I was probed for the *EAS* genes indicated. The result confirmed that *lpsB*, *dmaW*, *easH* and *easA* were on the same *Not*I fragment. The *lpsA* gene contains the only *Not*I site in the cluster, approximately 155 kb from the telomere, and the probe for *lpsA* was on the centromere side of that site. (C) DNA digested with *Not*I and probed first with a labeled segment of *lpsB*, followed by probing with a labeled telomere repeat array, or probed with the telomere array only, as indicated. (D) DNA digested with *Not*I was probed for the telomeric 6-module NRPS, or for *lpsB*, as indicated. The result indicated that the two genes are on the same telomeric *Not*I fragment.
(EPS)

Figure S3 Comparison of indole-diterpene synthesis (*IDT/LTM*) gene clusters in genomes of plant-associated Clavicipitaceae. Genes for synthesis of the skeleton compound, paspaline, are shown in blue, and genes for subsequent chemical decorations are shown in red. The function of *idtS/ltmS* (purple) is unknown. Identifiable genes flanking the clusters are indicated in gray. Open arrows and boxes indicate pseudogenes. Gray polygons between gene maps indicate gene orthologies, and gray arcs below the *E. festucae LTM* map indicate gene duplications giving rise to *ltmE* and *ltmJ*. Closed circles indicate telomeres, and distances from the telomeres are indicated in kilobasepairs (kb). Maps are arranged to illustrate synteny, and not to suggest an evolutionary history.
(EPS)

Figure S4 Synteny relationships between genes flanking non-telomeric alkaloid loci in Clavicipitaceae and orthologs in *Fusarium graminearum*. (A) Comparison of the regions flanking *EAS* in *C. purpurea* 20.1 with orthologous genes in *E. festucae* Fl1 and *F. graminearum* PH-1. (B) Comparison of the regions flanking *IDT* in *C. purpurea* 20.1 with orthologous genes in *E. festucae* Fl1 and *F. graminearum* PH-1. (C) Comparison of the regions flanking *LOL* in *E. festucae* E2368 with orthologous genes in *C. purpurea* 20.1 and *F. graminearum* PH-1. (D) Comparison of the regions flanking *perA* in *E. festucae* Fl1 with orthologous genes in *C. purpurea* 20.1 and *F. graminearum* PH-1. The *C. purpurea* genes are labeled with their gene names and, in parentheses, the gene identification numbers of their *F. graminearum* orthologs. Gray blocks indicate orthologous regions.
(EPS)

Figure S5 RIP-indices indicating repeat-induced point mutations in and near alkaloid loci. (A) *EAS* loci from *E. festucae* Fl1 and E2368. Gene names are abbreviated *A* through *H* for *easA* through *easH*, *W* for *dmaW*, and *clo* for *cloA*. (B) *IDT/LTM* loci from *E. festucae* Fl1 and E2368. Gene names are abbreviated *B* through *Q* for *ltmB* through *ltmQ.* (C) *LOL* locus and adjacent supercontigs from *E. festucae* E2368. Gene names are abbreviated *A* through *T* for *lolA* through *lolT*, and flanking genes *lteA* and *nsfA* are named. (D) *PER* loci from *E. festucae* Fl1 and E2368 and *E. typhina* E8 and E5819. Domains of *perA* are indicated as A (adenylation), T (thiolation), C (condensation), M (*N*-methylation) and R* (reduction). Subscripts indicate postulated specificity of adenylation domains for 1-pyrroline-5-carboxylate (A_P_) and arginine (A_R_). Tracks from top to bottom of each map represent the following: genes, graph of RIP index (ApT/TpA), repeats (cyan bars) and graphs of AT (red) and GC (blue) contents. Each gene is represented by a filled arrow indicating the direction of transcription, or in the case of (D) each gene is represented by one or more boxes representing the coding sequences in exons, and an arrow indicating the direction of transcription. Identifiable genes flanking the clusters are indicated in gray, and unfilled arrows indicate pseudogenes. Double-slash marks (//) indicate sequence gaps within the assembled scaffolds.
(EPS)

Table S1 Origins of isolates for which genomes were sequenced or survey-sequenced in this study.
(DOCX)

Table S2 Genome and sequence accession numbers. All data are in GenBank, except the *Claviceps purpurea* 20.1 assembly (76493), which is in the EMBL database.
(DOCX)

Table S3 Shimodaira-Hasegawa test results. Tree1 is the maximum likelihood estimate (MLE) tree obtained from the data. Δ*ln* L represents the difference between the MLE and likelihood value of Tree 2 under the model with the given data. The *p*-values are for the null hypothesis that Tree1 and Tree 2 are equally good explanations of the data for Tree1.
(DOCX)

Table S4 Secondary metabolism gene clusters in assembled C. purpurea and E. festucae genomes.
(DOCX)

Table S5 Summary of epichloae transposable elements identified within repeat regions. Abbreviations: *Eam = Epichloë amarillans, Ebe = E. brachyelytri, Efe = E. festucae, Egl = E. glyceriae, Ety = E. typhina, Nga = Neotyphodium gansuense, Ngi = N. gansuense* var. *inebrians*, retro-Tn = retrotransposon, Tn = transposon.
(DOCX)

Protocol S1 RIP-index perl script.
(PL) Click here for additional data file.

Protocol S2 Patch for OrthoMCL.
(PDF)
