# Supplementary material for: Plant-Symbiotic Fungi as Chemical Engineers: Multi-Genome Analysis of the Clavicipitaceae Reveals Dynamics of Alkaloid Loci
Source: PLoS Genet. 2013 Feb 28;9(2):e1003323. doi: 10.1371/journal.pgen.1003323 (PMC3585121; doi:10.1371/journal.pgen.1003323)
Supplement: Table S1 — Origins of isolates for which genomes were sequenced or survey-sequenced in this study. (DOCX) [file pgen.1003323.s006.docx]

**Table S1. Origins of isolates for which genomes were sequenced or survey-sequenced in this study.**

| Organism | Strain | Host | Geographical origin / Site of isolation (if different) | Collector | Reference^a^ |
| --- | --- | --- | --- | --- | --- |
| *Aciculosporium take* I. Miyake | MAFF-241224 | *Phyllostachys bambusoides* Siebold & Zucc. | Japan | Eiji Tanaka | [1] |
| *Claviceps fusiformis* Loveless | PRL 1980 | *Pennisetum typhoideum* Rich. | Cote d'Ivoire | V. E. Tyler, Jr. | [2] |
| *Claviceps paspali* F. Stevens & J.G. Hall | RRC-1481 | *Paspalum* L. sp. | Mansfield, Georgia, USA | Gretchen A. Kuldau | [3] |
| *Claviceps purpurea* (Fr.) Tul. | 20.1 | *Secale cereale* L. | Germany | Paul Tudzynski ^a^ | [4] |
| *Epichloë amarillans* J.F. White, Jr. | E57 | *Agrostis hyemalis* (Walter) Britton, Sterns & Poggenb. | Brazoria Co., Texas, USA | James F. White, Jr. | [5,6] |
| *Epichloë brachyelytri* Schardl & Leuchtm. | E4804 | *Brachyelytrum erectum* (Schreb.) P. Beauv. | Edmonson Co., Kentucky, USA | Leonardo J. Iannone | [7] |
| *Epichloë bromicola* Leuchtm. & Schardl | E502 | *Bromus erectus* Huds. | Ct. Vaud, Switzerland | A. Leuchtmann | [8] |
| *Epichloë elymi* Schardl & Leuchtm. | E56 | *Elymus virginicus* L. | Brazoria Co., Texas, USA | James F. White, Jr. | [7] |
| *Epichloë festucae* Leuchtm., Schardl & M.R. Siegel | E2368 | *Lolium* L. spp. | Europe / Lexington, Kentucky, USA | Christopher L. Schardl | [9] |
| *Epichloë festucae* Leuchtm., Schardl & M.R. Siegel | Fl1 | *Festuca trachyphylla* (Hack.) Krajina | Europe / Palmerston North, New Zealand | Michael J. Christensen | [10] |
| *Epichloë glyceriae* Schardl & Leuchtm. | E277 | *Glyceria striata* (Lam.) Hitchc. | Osgoode, Ottawa, Canada | Adrian Leuchtmann | [11] |
| *Epichloë glyceriae* Schardl & Leuchtm. | E2772 | *Glyceria striata* (Lam.) Hitchc. | Canastota, New York, USA | Adrian Leuchtmann | [11] |
| *Epichloë typhina* (Pers.) Tul. & C. Tul. | E8 | *Lolium perenne* L. | Europe / Massachusetts, USA | Noel Jackson | [5] |
| *Epichloë typhina* (Pers.) Tul. & C. Tul. | E5819 | *Poa nemoralis* L. | Zürich, Switzerland | Adrian Leuchtmann | [12,13] |
| *Neotyphodium gansuense* C.J. Li & Z.B. Nan | E7080 | *Achnatherum inebrians* (Hance) Keng | Sunan, Gansu, China | Chunjie Li | [14,15] |
| *N. gansuense* var. *inebrians* C.D. Moon, & Schardl | E818 | *Achnatherum inebrians* (Hance) Keng | Urumqi, Xinjiang, China | K.F. Min, I.  Fletcher, & P.S. Harris | [14] |
| *N. uncinatum* (W. Gams, O. Petrini & D. Schmidt) A.E. Glenn, C.W. Bacon & R.T. Hanlin | E167 | *Lolium pratense* (Huds.) Darbysh. | Nyon, Switzerland | Dorothea Schmidt | [16] |
| *Periglandula ipomoeae* U. Steiner, E. Leistner & Schardl | IasaF13 | *Ipomoea asarifolia* (Desr.) Roem. & Schult. | Ecuador / Bonn, Germany | Eckart Eich | [17] |

^a^ References:

1. Tanaka E, Tanaka C (2008) Phylogenetic study of clavicipitaceous fungi using acetaldehyde dehydrogenase gene sequences. Mycoscience 49: 115-125.

2. Pažoutová S, Tudzynski P (1999) *Claviceps* sp PRL 1980 (ATCC 26245), 59 and Pepty 695/ch-I: their true story. Mycological Research 103: 1044-1048.

3. Tooley PW, Goley ED, Carras MM, Frederick RD, Weber EL, et al. (2001) Characterization of Claviceps species pathogenic on sorghum by sequence analysis of the beta-tubulin gene intron 3 region and EF-1 alpha gene intron 4. Mycologia 93: 541-551.

4. Hüsgen U, Büttner P, Müller U, Tudzynski P (1999) Variation in karyotype and ploidy level among field isolates of *Claviceps purpurea*. Journal of Phytopathology 147: 591-597.

5. Siegel MR, Latch GCM, Bush LP, Fannin FF, Rowan DD, et al. (1990) Fungal endophyte-infected grasses: alkaloid accumulation and aphid response. Journal of Chemical Ecology 16: 3301-3315.

6. White JF, Jr. (1994) Endophyte-host associations in grasses.XX. Structural and reproductive studies of *Epichloë amarillans sp. nov.* and comparisons to *E. typhina*. Mycologia 86: 571-580.

7. Schardl CL, Leuchtmann A (1999) Three new species of *Epichloë* symbiotic with North American grasses. Mycologia 91: 95-107.

8. Leuchtmann A, Schardl CL (1998) Mating compatibility and phylogenetic relationships among two new species of *Epichloë* and other congeneric European species. Mycological Research 102: 1169-1182.

9. Wilkinson HH, Siegel MR, Blankenship JD, Mallory AC, Bush LP, et al. (2000) Contribution of fungal loline alkaloids to protection from aphids in a grass-endophyte mutualism. Molecular Plant-Microbe Interactions 13: 1027-1033.

10. Young CA, Bryant MK, Christensen MJ, Tapper BA, Bryan GT, et al. (2005) Molecular cloning and genetic analysis of a symbiosis-expressed gene cluster for lolitrem biosynthesis from a mutualistic endophyte of perennial ryegrass. Molecular genetics and genomics 274: 13-29.

11. Leuchtmann A, Clay K (1990) Isozyme variation in the *Acremonium/Epichloe* fungal endophyte complex. Phytopathology 80: 1133-1139.

12. Schardl CL, Leuchtmann A (2005) The epichloë endophytes of grasses and the symbiotic continuum. In: Dighton J, White JF, Oudemans P, editors. The Fungal Community: its organization and role in the ecosystem. 3rd ed. Boca Raton, Florida: CRC Press. pp. 475-503

13. Schardl CL, Leuchtmann A, McDonald BA (2007) Relationships of *Epichloë typhina* isolates from different host grasses. In: Popay A, Thom ER, editors. Proceedings of the 6th International Symposium on Fungal Endophytes of Grasses. Christchurch, New Zealand: New Zealand Grassland Association. pp. 451-455.

14. Moon CD, Guillaumin J-J, Ravel C, Li C, Craven KD, et al. (2007) New *Neotyphodium* endophyte species from the grass tribes Stipeae and Meliceae. Mycologia 99: 895-905.

15. Li CJ, Nan ZB, Paul VH, Dapprich PD, Liu Y (2004) A new *Neotyphodium* species symbiotic with drunken horse grass (*Achnatherum inebrians*) in China. Mycotaxon 90: 141-147.

16. Blankenship JD, Spiering MJ, Wilkinson HH, Fannin FF, Bush LP, et al. (2001) Production of loline alkaloids by the grass endophyte, *Neotyphodium uncinatum*, in defined media. Phytochemistry 58: 395-401.

17. Steiner U, Leibner S, Schardl CL, Leuchtmann A, Leistner E (2011) *Periglandula*, a new fungal genus within the Clavicipitaceae and its association with Convolvulaceae. Mycologia 103: 1133-1145.
