# Supplementary material for: Plant-Symbiotic Fungi as Chemical Engineers: Multi-Genome Analysis of the Clavicipitaceae Reveals Dynamics of Alkaloid Loci
Source: PLoS Genet. 2013 Feb 28;9(2):e1003323. doi: 10.1371/journal.pgen.1003323 (PMC3585121; doi:10.1371/journal.pgen.1003323)
Supplement: Table S2 — Genome and sequence accession numbers. All data are in GenBank, except the Claviceps purpurea 20.1 assembly (76493), which is in the EMBL database. (DOCX) [file pgen.1003323.s007.docx]

**Table S2. Genome and sequence accession numbers.**^a^

| Organism | Strain | Genome Project | *EAS* | *IDT/LTM* | *LOL* | *PER* |
| --- | --- | --- | --- | --- | --- | --- |
| *Aciculosporium take* | MAFF-241224 | PRJNA67241 |  | JN587272 |  |  |
| *Claviceps fusiformis* | PRL 1980 | PRJNA67243 | JN182233; JN186798 |  |  |  |
| *Claviceps paspali* | RRC-1481 | PRJNA51623 | JN186800 | JN613321; JN613322 |  |  |
| *Claviceps purpurea* | 20.1 | EMBL 76493  PRJEA76493 | JN186799 | JX402756 |  |  |
| *Epichloë amarillans* | E57 | PRJNA67301 |  |  | JF830812; JF830813 | JN640285 |
| *Epichloë brachyelytri* | E4804 | PRJNA67245 | JN378894; JN378895; JN378896 |  | JF800659; JF800661; JF800660 | JN613323 |
| *Epichloë bromicola* | E502 |  |  |  |  | JX441995 |
| Epichloë elymi | E56 | PRJNA173776 | JX439640; JX439641; JX439642 |  |  | JX402755 |
| *Epichloë festucae* | E2368 | PRJNA42133 | JN167225; JN167226; JN167227 | JX402753 | JF830815; JF830814; JF830816 | JN640287 |
| *Epichloë festucae* | Fl1 | PRJNA51625 | JN177500; JN177501; JN177502 | JN613318; JN613319; JN613320 |  | AB205145 |
| *Epichloë glyceriae* | E277 | PRJNA67247 | JN177503; JN177504; JN177505; JN177506 |  | JF800663; JF800664; JF800665 | JN640291 |
| *Epichloë typhina* | E8 | PRJNA174036 |  |  |  | JX402754 |
| *Epichloë typhina* | E5819 | PRJNA68441 | JN182230; JN182231; JN182232 |  |  | JN640289 |
| *Neotyphodium gansuense* | E7080 | PRJNA67299 |  | JN587271 | JF800666 | JN640290 |
| *N. gansuense* var. *inebrians* | E818 | PRJNA174039 | JX072969; JX273434 | JX072969 |  |  |
| *Neotyphodium uncinatum* | E167 |  |  |  | AY723749; AY723750; AY724686; JX430081; JX430082 |  |
| *Periglandula ipomoeae* | IasaF13 | PRJNA67303 | JN182229 | JN587270 |  |  |

^a^ All data are in GenBank, except the *Claviceps purpurea* 20.1 assembly (76493), which is in the EMBL database.
