# Supplementary material for: Plant-Symbiotic Fungi as Chemical Engineers: Multi-Genome Analysis of the Clavicipitaceae Reveals Dynamics of Alkaloid Loci
Source: PLoS Genet. 2013 Feb 28;9(2):e1003323. doi: 10.1371/journal.pgen.1003323 (PMC3585121; doi:10.1371/journal.pgen.1003323)
Supplement: Table S3 — Shimodaira-Hasegawa test results. Tree1 is the maximum likelihood estimate (MLE) tree obtained from the data. Δln L represents the difference between the MLE and likelihood value of Tree 2 under the model with the given data. The p-values are for the null hypothesis that Tree1 and Tree 2 are equally good explanations of the data for Tree1. (DOCX) [file pgen.1003323.s008.docx]

Table S3. Shimodaira-Hasegawa test results.^a^

| Tree 1 | Tree 2 | ∆*ln* L | *p*-value |
| --- | --- | --- | --- |
| *tefA* | *rpbA* | 835.6271 | <0.0001 |
| *tefA* | *rpbB* | 1057.2476 | <0.0001 |
| *rpbB* | *rpbA* | 24.98734 | 0.2138 |

**^a^** Tree1 is the maximum likelihood estimate (MLE) tree obtained from the data. ∆*ln* L represents the difference between the MLE and likelihood value of Tree 2 under the model with the given data. The *p*-values are for the null hypothesis that Tree1 and Tree 2 are equally good explanations of the data for Tree1.
