# Supplementary material for: Plant-Symbiotic Fungi as Chemical Engineers: Multi-Genome Analysis of the Clavicipitaceae Reveals Dynamics of Alkaloid Loci
Source: PLoS Genet. 2013 Feb 28;9(2):e1003323. doi: 10.1371/journal.pgen.1003323 (PMC3585121; doi:10.1371/journal.pgen.1003323)
Supplement: Table S4 — Secondary metabolism gene clusters in assembled C. purpurea and E. festucae genomes. (DOCX) [file pgen.1003323.s009.docx]

**Table S4. Secondary metabolism gene clusters in assembled *C. purpurea* and *E. festucae* genomes.**

| Cluster | Fungus | Cluster Type^a^ | Location | No. Genes | Comments | Repeat blocks |
| --- | --- | --- | --- | --- | --- | --- |
| 1 | *Cp-*20.1 | Alk, D, N | Supercontig22:235054..290780 | 13 | Ergot alkaloid biosynthesis (*EAS*) gene cluster. Genes for NRPS subunits LPS1 (*lpsA*1, *lpsA*2) (structures, A-T-C-A-T-C-A-T-C), LPS2 (*lpsB*) (A-T-C), LPS3 (*lpsC*) (A-T-C-R). | None |
|  | *Ef*-E2368 | Alk, D, N | Supercontig138:28978..62349, Supercontig306:1..17064, Supercontig248:2020..24284 | 11 | *EAS.* Subtelomeric in epichloae, but not other genera. Lysergyl peptide synthetase genes *lpsA* and *lpsB*. | Left, Inside, Right |
|  | *Ef-*Fl1 | Alk, D, N | Supercontig37:6449..85263 | 11 | " | " |
| 1a | *Cp-*20.1 | Alk, D | Supercontig22:344265-362253 | 3 | Contains two *dmaW* copies and one complete and one partial *easF* copy*.* | Inside |
| 2 | *Cp-*20.1 | Alk, T | Supercontig1:574647..587649 | 6 | Partial, nonfunctional indole-diterpene (*IDT*) gene cluster. | None |
|  | Ef-E2368 | Alk, T, D | Supercontig127:9180..22222 | 6 | Partial, nonfunctional IDT cluster. | Left, Inside, Right |
|  | *Ef-*Fl1 | Alk, T, D | Supercontig41:12004..89317 | 11 | *IDT*/ *lolitrem* (*LTM*) subtelomeric cluster. | " |
| 3 | Ef-E2368 | Alk | Supercontig 406:762..10003, Supercontig141:1.. 54515 | 11 | Loline (LOL) biosynthesis gene cluster. | Left, Inside, Right |
| 4 | *Ef*-E2368 | Alk, N | Supercontig49:158191..166609 | 1 | Peramine synthetase, *perA-∆R**, NRPS structure A-T-C-A-MT-T. Adjacent repeat block. | Left, Inside, Right |
|  | *Ef-*Fl1 | Alk, N | Supercontig13:332178..341421 | 1 | *perA* NRPS complete structure A-T-C-A-MT-T-R*. No large repeat block in Fl1. | " |
| 5 | *Cp-*20.1 | K | Supercontig35:273853..283997 | 2 | Lovastatin side chain/ T-toxin-related polyketide synthase (PKS). | None |
|  | *Ef*-E2368 | K | Supercontig2:117294..128577 | 3 | PKS structure KS–AT-KR-ACP. | None |
|  | *Ef-*Fl1 | K | Supercontig3:173547..184249 | 3 | " | " |
| 6 | *Cp-*20.1 | K | Supercontig63:140050..155625 | 2 | Lovastatin side chain/ T-toxin-related PKS. | None |
|  | *Ef*-E2368 | K | Supercontig20:12283..33175 | 4 | PKS structure KS–AT-DH-ER-KR-ACP. | None |
|  | *Ef-*Fl1 | K | Supercontig12:479617..500500 | 4 | " | " |
| 7 | *Ef*-E2368 | K | Supercontig33:210381..260006 | 14 | Equisetin/ lovastatin-related PKS. | Right to scaffold end |
|  | *Ef-*Fl1 | K | Supercontig68:5642..48892 | 14 | PKS structure KS-AT-DH-MT-ER-KR-ACP. | Left, Right |
| 8 | *Cp-*20.1 | K, T | Supercontig50:4995..37440 | 8 | Apparently derived from remnants of two clusters. PKS and some gibberellin biosynthesis gene homologs. | Inside |
|  | *Ef*-E2368 | K | Supercontig99:41460..68228 | 10 | Lovastatin side chain/ T-toxin-related PKS structure KS-AT-DH-ER-KR-ACP. | Right |
|  | *Ef-*Fl1 | K | Supercontig56:135341..162353 | 10 | " | Right |
| 9 | *Ef*-E2368 | K | Supercontig107:43116..53158 | 2 | PKS structure KS-AT-DH-MT-ER-KR-ACP. | Right |
| 10 | *Ef*-E2368 | K | Supercontig122**:**55198..59822 | 1 | Subtelomeric. Other dispensable genes. Lovastatin side chain/ T-toxin-related PKS. | None |
|  | *Ef-*Fl1 | K | Supercontig30:69213..84221 | 4 | PKS structure KS-ER-KR-ACP. | " |
| 11 | *Ef*-E2368 | K | Supercontig127:39899..60928 | 8 | Lovastatin PKS pseudogene structure KS-AT-DH-MT-ER-KR-ACP. Adjacent to *LTM.* | Left, Right |
|  | *Ef-*Fl1 | K | Supercontig41:90979..133665 | 8 | Repeat block in Fl1 separates PKS pseudogene from other cluster genes. | Left, Inside, Right |
| 12 | *Ef*-E2368 | K | Supercontig160:16607..32745 | 4 | Fumonisin-related PKS structure KS-AT-MT-KR-ACP. | Left, Right |
| 13 | *Ef*-E2368 | K | Supercontig183:18281..40277, Supercontig225:1..6400 | 7 | Repeat blocks in cluster. Fumonisin-related PKS structure KS-AT-DH-ER-KR-T. | Inside, Right |
|  | *Ef-*Fl1 | K | Supercontig46:28899..66074 | 7 | " | Left, Inside |
| 14 | *Cp-*20.1 | K | Supercontig62:93338..122072 | 6 | Pigment PKS structure KS-AT-DH-ACP-ACP-TE. Includes 3 tyrosinase homologs. | None |
| 15 | *Cp-*20.1 | K | Supercontig37:100311..133032 | 11 | Related to *Gibberella fujikuroi* FUM5 (fumonisin). PKS structure KS-AT-DH-MT-ER-KR-ACP. | None |
| 16 | *Cp-*20.1 | K | Supercontig43:227204..249517 | 3 | Related to pigment/ aflatoxin and 6-methylsalisylic acid. PKS structure KS-AT-DH-MT-ER-KR-ACP. | None |
| 17 | *Cp-*20.1 | K | Supercontig56:88272..97548 | 1 | Parolog of PKS 16, Cp-20.1 Supercontig43. | None |
| 18 | *Cp-*20.1 | K | Supercontig23:416820..425967 | 2 | PKS structure KS-AT-ACP. Related to pigment/ aflatoxin. | None |
| 19 | *Cp-*20.1 | K | Supercontig34:155941..181985 | 11 | PKS structure KS-AT-ACP. Related to pigment/ aflatoxin. | None |
| 20 | *Ef*-E2368 | K-N | Supercontig5:183162..213284 | 14 | PKS-NRPS structure KS–AT–DH-MT-ER-KR-ACP-C-A-T-R. | None |
|  | *Ef-*Fl1 | K-N | Supercontig9:395385..438374 | 13 | " | " |
| 21 | *Ef*-E2368 | K, D | Supercontig88:7179..45941 | 12 | Related to pigment/ aflatoxin PKS. | None |
|  | *Ef-*Fl1 | K, D | Supercontig23:48339..85481 | 11 | PKS structure NT-KS-AT-ACP-ACP-CYC-CYC. | " |
| 22 | *Ef*-E2368 | K, K | Supercontig45:116289..159277 | 9 | Pigment/ aflatoxin-related PKS, and Lovastatin side chain/ T-toxin-related PKS. | None |
|  | *Ef-*Fl1 | K, K | Supercontig45:29815..76531 | 10 | PKS structures KS–AT–ACP, and KS–AT–MT–KR-ACP-Carnitine acetyl transferase. | Left |
| 23 | *Ef*-E2368 | K, N | Supercontig25:251315..263521 | 2 | PKS gene structure KS-AT-DH-MT-KR-ER-PP. Nonribosomal peptide synthetase (NRPS) pseudogene. | Left |
| 24 | *Ef*-E2368 | K, N | Supercontig129:6950..32770 | 8 | Fum5-related PKS structure KS–AT–DH–MT–KR-ACP. NRPS structure A-T-C. | Left, Right |
|  | *Ef-*Fl1 | N | Supercontig41:196419..200539 | 2 | No PKS in this cluster in Fl1. | " |
| 25 | *Ef*-E2368 | N | Supercontig2:446887..476527 | 9 | NRPS structure A-T-C-R. | None |
|  | *Ef-*Fl1 | N | Supercontig3:500238..529891 | 9 | " | " |
| 26 | *Ef*-E2368 | N | Supercontig5:316183..318846 | 1 | NRPS pseudogene. | None |
|  | *Ef-*Fl1 | N | Supercontig9:274767..277429 | 1 | " | " |
| 27 | *Cp-*20.1 | N | Supercontig18:279329..300300 | 2 | NPS2-type, for intracellular siderophore. | None |
|  | *Ef*-E2368 | N | Supercontig6:382351..407973 | 5 | NRPS structure A-T-C-A-T-C-T-C-A-T-C-T-C-T-C. | None |
|  | *Ef-*Fl1 | N | Supercontig32:350041..369159 | 3 | " | Right |
| 28 | *Ef*-E2368 | N | Supercontig7:299131..312490 | 5 | NRPS pseudogene. | None |
|  | *Ef-*Fl1 | N | Supercontig26:154942..167933 | 3 | " | " |
| 29 | *Ef*-E2368 | N | Supercontig20:166524..186055 | 5 | NRPS structure A-T-E-C-A-T-C-A-T-C. | None |
|  | *Ef-*Fl1 | N | Supercontig12:638291..657761 | 5 | " | " |
| 30 | *Ef*-E2368 | N | Supercontig25:208732..219915 | 1 | NRPS structure C-A-T-E-C-A-T-C. Repeat block separates from PKS gene of another cluster. | Right |
| 31 | *Ef*-E2368 | N | Supercontig27:6755..70554 | 7 | 12-Module NRPS structure A-T-C-A-T-C-A-T-E-C-A-T-C-A-T-C-A-T-C-A-T-C-A-T-E-C-A-T-C-A-T-E-C-A-T-C-A-T-C-  -A-T-C-R. | Left to scaffold end |
|  | *Ef-*Fl1 | N | Supercontig58:135932..141422 | 2 | Fl1 lacks the NRPS, etc. | None |
| 32 | *Ef-*Fl1 | N | Supercontig58:143176..158380 | 6 | NRPS pseudogene. | Right |
| 33 | *Cp-*20.1 | N | Supercontig22:9468..55902 | 5 | 8-Module NRPS for extracellular siderophore. | Left, Inside |
|  | *Ef*-E2368 | N | Supercontig33:175761..218396 | 3 | NRPS structure A-T-C-A-T-C-A-T-E-C-A-T-C-A-T-E-C-A-T-E-C-A-T-C-A-T-C. | None |
|  | *Ef-*Fl1 | N | Supercontig10:513551..544501 | 3 | " | " |
| 34 | *Ef*-E2368 | N | Supercontig51:51854..55776 | 1 | NRPS structure A-T-R. | None |
|  | *Ef-*Fl1 | N | Supercontig25:131294..135688 | 1 | " | " |
| 35 | *Ef*-E2368 | N | Supercontig69:114963..140135 | 4 | *sidN* for epichloënin siderophores*.* Homolog of *sidC*. | Right to scaffold end |
|  | *Ef-*Fl1 | N | Supercontig11:301529..332774 | 4 | NRPS structure A-T-C-A-T-C-A-T-C-T-C-T-C.  Contains 6 kb AT-rich track. | None |
| 36 | *Ef*-E2368 | N | Supercontig155:10514..36612 | 3 | Fragments of cyclosporine synthetase (NRPS pseudogene), Repeat blocks in cluster. | Left, Inside |
| 37 | *Cp-*20.1 | N | Supercontig80:41972..59844 | 3 | Pseudogene. | None |
|  | *Ef*-E2368 | N | Supercontig158:32636..45185 | 2 | NRPS structure C-C-A-T-C. | None |
|  | *Ef-*Fl1 | N | Supercontig52:16476..28924 | 2 | " | None |
| 38 | *Ef*-E2368 | N | Supercontig167:12296..34006 | 7 | NRPS pseudogene. | None |
|  | *Ef-*Fl1 | N | Supercontig30:198901..219785 | 7 | " | Left |
| 39 | *Ef*-E2368 | N | Supercontig176:28705..44204, Supercontig229:3386..27297 | 8 | GliP-like NRPS but only one partial module. Repeat block in cluster in E2368, but not in Fl1. | Inside, Right |
|  | *Ef-*Fl1 | N | Supercontig1:79721..100754 | 8 | " | None |
| 40 | *Ef*-E2368 | N | Supercontig194:10557..36717 | 1 | NRPS structure T-C-A-T-E-C-A-T-E-C-A-T-C-A-T-C-A-T-E-C-A-T-C. Subtelomeric. | Left, Inside |
| 41 | *Cp-*20.1 | N | Supercontig31:335724..355415 | 5 | For extracellular siderophore. | None |
| 42 | *Cp-*20.1 | N | Supercontig6:182887..188084 | 1 | NRPS structure A-T-R-KR. | None |
|  | *Ef*-E2368 | N | Supercontig86:47579..52233 | 1 | NRPS structure A-T-R-KR. | None |
|  | *Ef-*Fl1 | N | Supercontig56:43877..48271 | 1 | " | " |
| 43 | *Cp-*20.1 | N, N | Supercontig46:104332..139048 | 2 | NRPS structure A-T-C-A-T-C-A divergently transcribed from NRPS T-C-A-T-C-A-T-E-C-A-T-C-A-T-C-A-T-C. | None |
| 44 | *Cp-*20.1 | N, D | Supercontig12:359916..384628 | 11 | Epipolythiodioxopiperazine (*ETP*) gene cluster. | None |
|  | *Ef*-E2368 | N, D | Supercontig45:169668..194474 | 10 | NRPS structure A-T-C-A-T-C. | None |
|  | *Ef-*Fl1 | N, D | Supercontig73:22295..44905 | 9 | " | Right |
| 45 | *Ef*-E2368 | T | Supercontig137:53985..64572, Supercontig242:1..25563, Supercontig413:1..9799, Supercontig159:1..34751 | 7 | Very large, AT-rich repeat blocks throughout cluster. | Left, Inside, Right |
|  | *Ef-*Fl1 | T | Supercontig5:328587..450094 | 7 | " | " |
| 46 | *Cp-*20.1 | T | Supercontig27:173435..191089 | 4 | Some genes related to gibberellin (GA) synthesis genes. | Left, Inside, Right |
| 47 | *Cp-*20.1 | D | Supercontig10:609489..634228 | 8 | NRPS-like gene present. | None |
| 48 | *Cp-*20.1 | Misc. | Supercontig30:144318..156784 | 5 | PKS-like. | None |
| 49 | *Ef*-E2368 | Misc. | Supercontig39:212379..222984 | 5 | None of the signature genes. | Right |
|  | *Ef-*Fl1 | Misc. | Supercontig38:210261..219748 | 5 | " | Right |
| 50 | *Ef*-E2368 | Misc. | Supercontig166:10833..28346 | 7 | Subtelomeric and *recQ*-linked (left). | None |
|  | *Ef-*Fl1 | Misc. | Supercontig30:23729..44609 | 7 | " | Left, Right |
| 51 | *Cp-*20.1 | Misc. | Supercontig30:144318..156784 | 5 | NRPS-like. | None |

**^a^** Cluster types listed as alkaloid (Alk) and, by signature genes, polyketide synthase (K), nonribosomal peptide synthetase (N), K-N and N-K hybrids, DMATS-family prenyltransferase (D), terpene cyclase (T), and miscellaneous (Misc.).
