# Supplementary material for: Plant-Symbiotic Fungi as Chemical Engineers: Multi-Genome Analysis of the Clavicipitaceae Reveals Dynamics of Alkaloid Loci
Source: PLoS Genet. 2013 Feb 28;9(2):e1003323. doi: 10.1371/journal.pgen.1003323 (PMC3585121; doi:10.1371/journal.pgen.1003323)
Supplement: Table S5 — Summary of epichloae transposable elements identified within repeat regions. Abbreviations: Eam = Epichloë amarillans, Ebe = E. brachyelytri, Efe = E. festucae, Egl = E. glyceriae, Ety = E. typhina, Nga = Neotyphodium gansuense, Ngi = N. gansuense var. inebrians, retro-Tn = retrotransposon, Tn = transposon. (DOCX) [file pgen.1003323.s010.docx]

**Table S5. Summary of epichloae transposable elements identified within repeat regions.^a^**

|  | Species & Strain | *Eam* E57 | *Ebe* E4804 | *Eel* E56 | *Efe* E2368 | *Efe* Fl1 | *Egl* E277 | *Ety* E8 | *Ety* E5819 | *Ngu* E7080 | *Ngi* E818 |
| --- | --- | --- | --- | --- | --- | --- | --- | --- | --- | --- | --- |
| Repeat | **Class** |  |  |  |  |  |  |  |  |  |  |
| 1/Tahi | Class I retro-Tn |  |  |  | + | + |  |  |  |  | + |
| 2/Rua | Class I retro-Tn | + | + | + | + | + | + | + | + | + | + |
| 4/Wha | Class II DNA Tn, putative Mutator |  |  |  | + |  |  |  |  |  |  |
| 6/Ono | Class I retro-Tn |  | + |  | + | + | + | + | + | + |  |
| 7/Whitu | Class I retro-Tn |  |  |  | + | + |  | + | + |  |  |
| 8/Waru | Class II DNA Tn, putative Pif/Harbinger |  |  |  | + | + |  | + | + | + | + |
| 11 | Class I retro-Tn | + | + |  | + | + | + | + | + |  |  |
| 12 | Class I retro-Tn, putative |  |  |  | + | + |  | + | + | + | + |
| 13 | Class I retro-Tn, putative | + | + |  | + | + |  | + | + | + | + |
| 14 | Class I retro-Tn, putative Copia | + | + | + | + | + | + | + |  |  |  |
| 15 | Class I retro-Tn, putative Gypsy | + | + | + | + | + | + | + | + | + | + |
| 16 | Class I retro-Tn, putative |  | + | + | + | + | + | + | + | + | + |
| 17 | Class I retro-Tn |  | + |  | + | + |  | + | + | + | + |
| 18 | Class I, putative LINE | + | + | + | + | + | + | + | + | + |  |
| 19 | Class I retro-Tn, putative | + | + |  | + | + | + | + |  | + |  |
| 20 | Class I, putative LINE | + | + | + | + | + | + | + | + |  |  |
| 21 | Class II Putative DNA/hAT-Ac element |  |  |  | + | + |  |  | + |  |  |
| 22 | undefined |  |  |  | + |  |  |  |  |  | + |
| 23 | Class I retro-Tn | + |  |  | + |  |  |  |  |  |  |
| 24 | Class I retro-Tn |  |  |  | + |  |  |  |  | + | + |
| 25 | undefined |  |  |  | + |  |  |  |  |  |  |
| 26 | undefined |  |  |  | + |  |  |  | + |  |  |
| 27 | undefined |  |  | + | + | + | + | + |  | + |  |
| 28 | Class I retro-Tn, putative Copia | + | + |  | + | + |  | + |  | + | + |
| 29 | undefined |  |  |  | + |  |  | + |  |  |  |
| 30 | Class I retro-Tn | + | + | + |  |  | + | + | + | + | + |
| 31 | Class I retro-Tn | + | + | + |  |  | + | + | + | + | + |
| 32 | undefined |  | + |  | + | + |  | + |  |  |  |
| 33 | undefined |  |  |  |  |  |  | + |  |  |  |
| 34 | Class I retro-Tn |  |  |  |  |  |  | + |  |  |  |
| 35 | Class I retro-Tn | + | + | + |  |  | + | + | + |  | + |
| 36 | Class I retro-Tn | + | + | + |  |  |  |  |  |  | + |
| 37 | Class I retro-Tn |  | + |  |  |  |  |  |  |  |  |
| 38 | Class I retro-Tn |  | + |  |  |  | + |  |  |  |  |
| 40 | undefined |  |  | + |  |  |  |  |  |  |  |
| 41 | Class II DNA Tn, putative Tc1/mariner |  |  |  |  |  |  |  |  | + | + |
| 42 | Class II DNA Tn |  |  |  |  |  |  |  |  | + | + |
| 43 | undefined |  |  |  |  |  |  |  |  |  | + |
| 44 | Class II DNA Tn |  |  |  |  |  |  |  |  |  | + |
| 45 | undefined |  |  |  |  |  |  |  |  | + |  |
| 46 | undefined |  |  |  |  |  |  |  |  |  | + |
| 47 | undefined |  |  |  |  |  |  |  |  |  | + |
| 48 | undefined |  |  |  |  |  |  |  |  |  | + |

^a^Abbreviations: *Eam =* *Epichloë amarillans, Ebe = E. brachyelytri, Efe = E. festucae, Egl = E. glyceriae, Ety = E. typhina, Nga = Neotyphodium gansuense, Ngi = N. gansuense* var. *inebrians*, retro-Tn = retrotransposon, Tn = transposon.
