# Supplementary material for: Plant-Symbiotic Fungi as Chemical Engineers: Multi-Genome Analysis of the Clavicipitaceae Reveals Dynamics of Alkaloid Loci
Source: PLoS Genet. 2013 Feb 28;9(2):e1003323. doi: 10.1371/journal.pgen.1003323 (PMC3585121; doi:10.1371/journal.pgen.1003323)
Supplement: Protocol S2 — Patch for OrthoMCL. (PDF) [file pgen.1003323.s012.pdf]

Protocol S1. Fixes for COCO-CL including use of MUSCLE for multiple sequence alignment.

```
#MUSCLE-and-fixes-for-COCO-CL

diff -ur ../../coco-cl.orig/coco-cl.cpp ./coco-cl.cpp
--- ../../coco-cl.orig/coco-cl.cpp	2006-06-08 17:21:06.000000000 -0400
+++ ./coco-cl.cpp	2011-09-12 11:33:30.000000000 -0400
@@ -159,6 +159,9 @@
    {
     mid = (high+low)/2;                // initially 0.0

+    if (high == mid || low == mid)      // The binary search is as close as
possible.
+      break;
+
     for(int i = 1; i <= f1; i++) // Resetting the group Ids (cluster #) of all
proteins to be 0
       proteinInGroup[i]= 0;

diff -ur ../../coco-cl.orig/bootstrap.pl ./bootstrap.pl
--- ../../coco-cl.orig/bootstrap.pl	2006-06-08 17:06:32.000000000 -0400
+++ ./bootstrap.pl	2011-09-12 11:28:31.000000000 -0400
@@ -56,14 +56,16 @@
 # Variables related to boostrap alignments, dst, cluster, etc files (temporary)
 $bootstrapAln = $ARGV[0];   # sampled bootstrap alignment
 $bsFastaAln = $ARGV[0];          # sampled bootstrap alignment file in Fasta
format (for Clustalw)
+$bsFastaAlnPp = $ARGV[0];   # preprocessed version for Muscle
 $scriptFile = $ARGV[0];          # file containing Clustalw input commands for
the temp bootstrap aln
-$tempDst = $ARGV[0];        # file containing bootstrap temporary distance
matrix
+$tempDst = $ARGV[0];        # file containing bootstrap temporary distance
matrix (clustal format)
 $tempCls = $ARGV[0];        # file containing bootstrap temporary cluster
details
 $tempPh= $ARGV[0];          # file containing bootstrap temporary tree
 $tempNet = $ARGV[0];        # file containing bootstrap temporary Pajek graph
network

 substr($bootstrapAln, -3, 3) = "bootstrap";
 substr($bsFastaAln, -3, 3) = "temp.aln";
+substr($bsFastaAlnPp, -3, 3) = "temp.alnpp";
 substr($scriptFile, -3, 3) = "script";
 substr($tempDst, -3, 3) = "temp.dst";
 substr($tempCls, -3, 3) = "temp.cls";
@@ -141,12 +143,15 @@
   close ALN;

   # writing clustalw options (to be read by clustalw) onto the scriptFile
-  open(SCRIPT, ">$scriptFile") || die "can't open file $scriptFile"; #file
with .script extn
```

```
-  print SCRIPT "4\n1\n$bsFastaAln\n6\n3\n\n4\n\n\n\nx\n\n";
-  close SCRIPT;
+  # open(SCRIPT, ">$scriptFile") || die "can't open file $scriptFile"; #file
with .script extn
+  # print SCRIPT "4\n1\n$bsFastaAln\n6\n3\n\n4\n\n\n\nx\n\n";
+  # close SCRIPT;

   # Running clustalw on the boostrap aln file to generate .dst & .ph (phylip
tree) file
-  system("clustalw < $scriptFile");
+  # system("clustalw < $scriptFile");
+
+  # NFM 2011-04-20 - use clustalw non-interactively.
+  system "clustalw", "-infile=$bsFastaAln", "-tree", "-outputtree=dist";

   # Running coco-cl on the .dst file (distance matrix for the bootstrap aln)
   system("./coco-cl $tempDst");
@@ -251,13 +256,13 @@
 $bootstrapScore = $sum_alpha/$ARGV[2];       # average 'alpha' of all bootstrap
alignments

 # deleting all the temporary files that were generated
-system("rm $bootstrapAln");
-system("rm $bsFastaAln");
-system("rm $scriptFile");
-system("rm $tempDst");
-system("rm $tempCls");
-system("rm $tempPh");
-system("rm $tempNet");
+system("rm $bootstrapAln") unless exists $ENV{CCL_KEEP_TEMP};
+system("rm $bsFastaAln") unless exists $ENV{CCL_KEEP_TEMP};
+# system("rm $scriptFile") unless exists $ENV{CCL_KEEP_TEMP};
+system("rm $tempDst") unless exists $ENV{CCL_KEEP_TEMP};
+system("rm $tempCls") unless exists $ENV{CCL_KEEP_TEMP};
+system("rm $tempPh") unless exists $ENV{CCL_KEEP_TEMP};
+system("rm $tempNet") unless exists $ENV{CCL_KEEP_TEMP};


 #################################################################
diff -ur ../../coco-cl.orig/recurse.pl ./recurse.pl
--- ../../coco-cl.orig/recurse.pl 2006-06-08 17:06:32.000000000 -0400
+++ ./recurse.pl 2011-04-27 12:49:00.000000000 -0400
@@ -26,14 +26,12 @@
   die "C - number of bootstraps to be tried\n";
 }

-system("./runClustalW.pl $ARGV[0]");
+system("./runMuscle.pl $ARGV[0]");

 @words = split /\./, $ARGV[0];
 $dstFile = $words[0].".dst";
 $phFile = $words[0].".ph";
```

```
 $fastaFile = $words[0].".fasta";
-$dndFile = $words[0].".dnd";
-system("rm $dndFile");

 system("./fasta2seqrows.pl $fastaFile");
 $seqrowsFile = $words[0].".seqrows";
diff -ur ../../coco-cl.orig/runClustalW.pl ./runClustalW.pl
--- ../../coco-cl.orig/runClustalW.pl   2006-06-08 17:06:32.000000000 -0400
+++ ./runClustalW.pl   2011-04-21 13:30:40.000000000 -0400
@@ -6,17 +6,24 @@
   die "USAGE: runClustalW.pl <file containing sequences in FASTA format>\n\n";
 }

-$clustalOptionsFile = $ARGV[0].".cwScript";
+# $clustalOptionsFile = $ARGV[0].".cwScript";
 $inputFile = $ARGV[0];

-open(CLUSTALscript, ">$clustalOptionsFile") || die "can't open file
$clustalOptionsFile";
-
-print CLUSTALscript
"1\n$inputFile\n2\n9\n1\nF\n\n1\n\n\nX\n\n4\n6\n3\n\n4\n\n\n\nX\n\n";
-
-close(CLUSTALscript);
-
-system("clustalw < $clustalOptionsFile");
-
-system("rm $clustalOptionsFile");
-
-
+# open(CLUSTALscript, ">$clustalOptionsFile") || die "can't open file
$clustalOptionsFile";
+#
+# print CLUSTALscript
"1\n$inputFile\n2\n9\n1\nF\n\n1\n\n\nX\n\n4\n6\n3\n\n4\n\n\n\nX\n\n";
+#
+# close(CLUSTALscript);
+#
+# system("clustalw < $clustalOptionsFile");
+#
+# system("rm $clustalOptionsFile") unless exists $ENV{CCL_KEEP_TEMP};
+
+# NFM 2011-04-20 - use clustalw non-interactively.
+# Run once to do the alignment, once to get a tree.
+
+# Output file should always be .fasta.
+my $outputFile = $inputFile;
+$outputFile =~ s/[^.]*$/fasta/;
+system "clustalw", "-infile=$inputFile", "-align", "-output=FASTA", "-
outfile=$outputFile";
+system "clustalw", "-infile=$outputFile", "-tree", "-outputtree=dist";
diff -ur ../../coco-cl.orig/runMuscle.pl ./runMuscle.pl
```

```
--- ../../coco-cl.orig/runMuscle.pl	1969-12-31 19:00:00.000000000 -0500
+++ ./runMuscle.pl	2011-09-12 11:30:47.000000000 -0400
@@ -0,0 +1,63 @@
+#!/usr/bin/perl -w
+
+use strict;
+
+my $numArgs = $#ARGV + 1;
+if($numArgs != 1)
+{
+  die "USAGE: runMuscle.pl <file containing sequences in FASTA format>\n\n";
+}
+
+my $in_seqs = $ARGV[0];
+
+
+# Output file should always be .fasta.
+my $base = $in_seqs;
+$base =~ s/\.[^.]*$//;
+my $im_seqs = "$base.2.fasta";
+
+# Preprocess input FASTA by replacing names with strings unique in the
+# first 10 characters.  This is necessary for deciphering muscle's
+# matrix output.  This also avoids problems with using the same
+# file for input and output.
+my $i=0;
+my @seqids = ();
+open SEQ_IN, "<", $in_seqs;
+open SEQ_OUT, ">", $im_seqs;
+while(<SEQ_IN>) {
+	chomp;
+	if (/^>(.*)/) {
+		$_ = sprintf ">seq%06d %s", $i, $1;
+		$seqids[$i] = $1;
+		++$i;
+	}
+	print SEQ_OUT "$_\n";
+}
+close SEQ_IN;
+close SEQ_OUT;
+
+my $out_matrix = "$base.dst";
+
+my $im_align = "$base.faln";
+my $out_align = "$base.fasta";
+
+# Generate the .faln FASTA alignment and the .mtx matrix.
+system "muscle",  "-fasta", "-in", $im_seqs, "-out", $im_align;
+
+unlink $im_seqs unless exists $ENV{CCL_KEEP_TEMP};
+
+# Move the .faln to $out_align; we couldn't use that as the -out parameter,
```

```
+# because it may have been the same as $in_seqs.
+open ALN_IN, "<", $im_align;
+open ALN_OUT, ">", $out_align;
+while(<ALN_IN>) {
+     chomp;
+     s/^>seq\d+\s+/>/;
+     print ALN_OUT "$_\n";
+}
+close ALN_IN;
+close ALN_OUT;
+unlink $im_align unless exists $ENV{CCL_KEEP_TEMP};
+
+system "clustalw", "-infile=$out_align", "-tree", "-outputtree=dist";
+__END__
```
